# The Role of Meprins on the Brain Extracellular Matrix and Perineuronal Nets

**DOI:** 10.1096/fj.202601333R

**Published:** 2026-07-02

**Authors:** Simon Kreiselmaier, Maximilian Keller, Leonardo Nardi, Constantin Mueller, Nele von Wiegen, Kira Bickenbach, Lea Gröbner, Mohammad Abukhalaf, Andreas Tholey, Margareta M. Mueller, Hagen Körschgen, Michael J. Schmeisser, Christoph Becker‐Pauly, Claus U. Pietrzik

**Affiliations:** ^1^ Institute for Pathobiochemistry University Medical Center Mainz Mainz Germany; ^2^ Molecular Neurodegeneration, Institute for Pathobiochemistry University Medical Center Mainz Mainz Germany; ^3^ Institute of Anatomy University Medical Center Mainz Mainz Germany; ^4^ Unit for Degradomics of the Protease Web, Institute of Biochemistry Christian‐Albrechts‐Universität Zu Kiel Kiel Germany; ^5^ Systematic Proteome Research & Bioanalytics, Institute for Experimental Medicine Christian‐Albrechts‐Universität Zu Kiel Kiel Germany; ^6^ Molecular Cell Biology Laboratory, Institute of Technical Medicine Hochschule Furtwangen University Villingen‐Schwenningen Germany

**Keywords:** brevican, memory consolidation, meprin α, meprin β, neurocan, perineuronal nets, RPTP‐zeta, synapses

## Abstract

Meprin α and meprin β are zinc metalloproteases that are strongly expressed in intestinal and renal tissues and are expressed as homo‐ and heterodimers. In the kidney and intestine, they are involved in extracellular matrix assembly and modulation of inflammatory responses. However, meprin β has recently attracted attention because it generates Alzheimer's Disease (AD)‐specific Aβ peptides and cleaves brevican, a major component of the perineuronal nets (PNNs) in the brain. PNNs stabilize synapses, thereby regulating plasticity and memory formation. Brevican cleavage correlated with impaired spatial memory formation and impaired CA1 long‐term potentiation (LTP) in meprin β transgenic mice. Furthermore, numerous studies have shown the dysregulation of PNN components in AD. Still, the physiological and pathological functions of proteolytic PNN remodeling remain elusive. This study identified an essential role of meprin α in brevican cleavage. It enhanced meprin β's catalytic activity on brevican in co‐expression. Moreover, an N‐terminomics analysis identified novel meprin β substrates, neurocan, and receptor‐type tyrosine‐protein phosphatase zeta (RPTPζ) in the brain. Both are key components of PNNs. RPTPζ cleavage by meprin α and meprin β was confirmed in vitro. To assess the functional impact of meprin‐mediated proteolysis on the brain extracellular matrix, PNNs and synaptic organization were investigated in vivo using immunofluorescence and electron microscopy. Meprin‐mediated proteolysis disrupted PNN structure and decreased synapse density in the hippocampal CA1 region of meprin β transgenic mice. This identifies meprin‐dependent PNN remodeling as a novel mechanism contributing to synaptic dysfunction.

## Graphical abstract

Meprin β overexpression is implicated in Alzheimer's Disease. Recent studies identified brevican as a meprin β substrate, and this study demonstrates that meprin α co‐expression enhances meprin β‐mediated brevican cleavage. Further, neurocan and RPTPζ were identified as novel meprin β substrates in an N‐terminomics analysis of meprin β transgenic mice, while RPTPζ was additionally processed by meprin α in vitro. Meprin‐mediated proteolysis of these perineural net components impaired perineural net structure and reduced hippocampal CA1 synapse density in vivo.

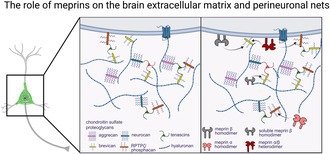

## 1 Introduction

Memory loss in health and disease, and its molecular and cellular basis is one of the most captivating questions in modern neurosciences [1, 2]. To date, investigations into the molecular and cellular basis of memories have revealed multiple intracellular and extracellular processes that modulate their formation, consolidation, and storage. However, the mechanisms involved remain incompletely understood [2]. One process that affects the formation and storage of long‐term memories is the formation of perineuronal nets (PNNs). PNNs are condensed extracellular matrix (ECM) structures that predominantly envelop GABAergic parvalbumin‐positive interneurons (PV+) in the cortex and hippocampus [3, 4, 5]. In neuronal development, they appear in close correlation with the closure of the critical period, thereby limiting juvenile plasticity and enwrapping neurons and synapses in lattice‐like structures [6, 7, 8]. PNNs are composed of chondroitin sulfate proteoglycans (CSPGs), including brevican, aggrecan, neurocan, as well as connective proteins such as receptor‐type tyrosine‐protein phosphatase zeta (RPTPζ), hyaluronan and proteoglycan link protein 1 (HAPLN1), tenascin‐R and ‐C, and hyaluronic acid [3, 4, 9, 10].

PNNs play a crucial role in memory formation and synaptic plasticity, as they were proposed to structurally stabilize synaptic contacts [1]. To date, studies on the role of the ECM in memory and learning have primarily focused on chondroitinase ABC (ChABC)‐mediated digestion of chondroitin sulfate (CS) side chains that are physiologically bound to proteoglycans in PNNs. This catalytic process was shown to inhibit auditory fear‐conditioned memory formation [11] and to impair the acquisition and reconsolidation of cocaine‐induced place preference memories [12]. Furthermore, ChABC‐mediated digestion was reported to enable the erasure of fear‐conditioned memories in adult mice, a process that is physiologically only seen in juvenile animals [13].

To assess the effects of chondroitin sulfate proteoglycans on memory, several proteoglycan‐knockout mouse models were generated. Brevican‐ and neurocan‐knockout mice exhibited impaired hippocampal CA1 long‐term potentiation, a synaptic process crucial for learning [14, 15]. Additionally, brevican‐ and RPTPζ‐knockout mice showed structurally impaired PNNs [14, 16]. Further studies on the effect of brevican on synaptic plasticity revealed that brevican modulates the α‐amino‐3‐hydroxy‐5‐methyl‐4‐isoxazolepropionic acid receptor (AMPAR) and potassium channel location at synapses, thus also affecting short‐term synaptic plasticity [17]. Initiated by this brevican‐mediated link between synaptic plasticity and PNN structure, the effects of PNNs on learning and memory are a subject of research in various neuropsychiatric diseases, ranging from neurodevelopmental to neurodegenerative diseases such as Alzheimer's Disease (AD) [5, 18, 19].

Given that PNN composition and integrity are dynamically regulated, increasing attention has been directed to ECM modifying enzymes that may influence remodeling and, consequently, neuronal plasticity [5]. Among these enzymes, metalloproteases represent an important class of regulators capable of processing ECM components and associated proteins [20]. Despite the discovery of the metalloproteases meprin α and meprin β over four decades ago, the full extent of their physiological functions is still incompletely understood. Meprin α and meprin β function as zinc‐dependent metalloproteases that belong to the astacin family [21]. They were first discovered in mouse renal and intestinal tissues and were shown to play a role in tissue repair and modulation of inflammatory responses [22, 23, 24, 25]. Subsequent studies revealed their expression in a variety of additional tissues [26, 27, 28, 29, 30, 31, 32, 33]. They were identified as susceptibility genes in inflammatory bowel disease, acute kidney injury, and diabetic nephropathy, and were shown to be involved in the remodeling of the skin's ECM [27, 34, 35, 36, 37, 38, 39]. Well‐known substrates of meprin β include E‐cadherin, galectin 3, procollagen III, mucin 2, and APP [26, 27, 28, 29, 30, 31, 32, 33]. Recently, more attention has been directed toward the function of meprins in the brain with the discovery of meprin β overexpression in Alzheimer's Disease (AD) [40, 41], and the meprin β‐mediated generation of N‐terminally truncated Aβ1‐40 and Aβ2‐40 peptides from APP [30, 31, 32]. In contrast to meprin β, considerably less is known about the role of meprin α in the brain. However, one identified substrate of meprin α in cerebellar granular neurons is reelin, a large secreted glycoprotein that plays an important role in regulating synaptic plasticity [42, 43].

Notably, meprin β and meprin α, unlike other ECM proteases, have a striking specificity for negatively charged amino acids glutamate (E, amino acid in single letter code) and aspartate (D) in positions C‐terminal of the cleavage site [44]. Although they share 55% amino acid sequence identity within their protease domain and exhibit a similar cleavage specificity, a key structural difference lies in the unique ‘inserted' domain’ in meprin α, which contains a furin cleavage site (Figure S4A) [44]. This inserted domain enables meprin α to undergo furin‐mediated cleavage in the Golgi, resulting in a secreted form (Figure S4B) [44]. In contrast, meprin β predominantly remains membrane‐bound but can be shed by ADAM10/17 in its inactive form (Figure S4B) [45]. Extracellularly, the zymogens of both meprin α and meprin β subunits are activated through cleavage of their N‐terminal propeptide by proteases with trypsin‐like activity, such as matriptase‐2 [45, 46]. Both meprin α and meprin β physiologically form homodimers [37]. Secreted meprin α homodimers are known to oligomerize extracellularly, forming large protease complexes in the mega‐Dalton range [37, 47]. Interestingly, both murine and human meprin α and meprin β subunits have been shown to be capable of heterodimerization [48, 49]. These heterodimers, also referred to as meprin α/β heterodimers, result in the meprin α subunits being anchored to the cell surface through the meprin β subunit (Figures S4B and S5) [49]. These localization differences between secreted meprin α and membrane‐associated meprin β were hypothesized to influence their respective substrate specificity and biological function. Intriguingly, heterodimerization was shown to enhance the catalytic activity of both meprin α and meprin β subunits in a fluorogenic activity assay [49].

Most recently, a transgenic mouse model was created that exclusively overexpresses meprin β in neurons of the hippocampus and cortex, to further investigate the role of meprins in the central nervous system [50]. These mice exhibited significant impairments in spatial learning and long‐term potentiation (LTP) that could not be attributed to APP processing [50]. Using a High‐Efficiency Undecanal‐based N‐Termini EnRichment (HUNTER) N‐Terminomics analysis, brevican was identified as a novel meprin β substrate, likely contributing to the observed phenotype [50]. In this study, we investigated the role of meprin α in brevican cleavage. Moreover, we examined neurocan and RPTPζ as probable novel proteolytic substrates of meprin β, whose proteolysis may also contribute to the observed phenotype in meprin β‐overexpressing animals. Since these proteins are key components of PNNs, we investigated the effect of meprins on the structure of the brain ECM, PNNs, and synapses.

## 2 Methods

### 2.1 Animals

A mouse strain was generated that exclusively overexpressed meprin β in hippocampal and cortical neurons using a NEX driver. For this, NEX^Cre;TG/wt^ mice [51] and Rosa26^Mep1b‐HA^ mice [52] were crossed, resulting in a mepβ^Cre;TG/wt^ and mepβ^Cre;wt/wt^ genotype [50]. The mice were kept on a 12 h light/dark cycle and had access to food and water *ad libitum*. All procedures involving animals were carried out in accordance with European and German guidelines for the care and use of laboratory animals, and they were approved by the Central Animal Facility of the University of Mainz and the ethical committee on animal care and use of Rhineland–Palatinate, Germany (G20‐1‐026). In total, 12 mice of mixed gender were used for the immunofluorescence and N‐terminomics experiments.

### 2.2 HUNTER N‐Terminomics Analysis

For N‐terminome analysis, the HUNTER workflow was employed [53] (*n* = 3 per genotype). To dissolve the precipitated proteins, 1 mL of buffer containing 1% SDS and 50 mM HEPES (pH 8), supplemented with cOmplete EDTA‐free protease inhibitor cocktail (1 mg/mL; Thermo, Waltham, Massachusetts, USA), was used. Individual protein concentrations were quantified using the Pierce BCA Protein Assay Kit (23 225 Thermo, Waltham, Massachusetts, USA). Each sample (100 μg protein) was subjected to reduction and alkylation with 24 mM TCEP and 80 mM chloroacetamide for 1 h at 25°C in the dark. Protein purification was carried out using SP3 beads at a 1:20 (w/w) protein‐to‐bead ratio, with incubation for 15 min at 25°C in a solution containing 50% ethanol. The supernatant was discarded, and the beads were rinsed three times with 80% ethanol. For subsequent TMT labelling, the beads were resuspended in 30 μL of 0.5 M HEPES (pH 8), 22.5 μL of 6 M guanidine hydrochloride, and 4.5 μL of 125 mM TCEP, followed by incubation for 30 min at 25°C. Isobaric tandem mass tags (14.04 mg/mL TMTsixplex reagents; Thermo, Waltham, Massachusetts, USA) were dissolved in ethanol for 5 min while being gently vortexed. Bead‐bound samples were labeled according to the manufacturer's protocol. Labeling reactions were carried out on the SP3 beads for 1.5 h at 25°C in the dark, after which 8 μL of 8% hydroxylamine was added and incubated for 30 min at 25°C to quench the reaction. For sample cleanup, each group of labeled samples was combined, mixed with ethanol to reach 80%, and incubated for 15 min at 25°C. The beads were washed three times with 80% ethanol, centrifuged to eliminate residual liquid, and reconstituted in 200 mM HEPES buffer (pH 8). Digestion was carried out overnight at 37°C using trypsin at a 1:50 enzyme‐to‐protein ratio. A 10 μL aliquot of the supernatant (equivalent to 15 μg of protein) was retained as the pre‐HUNTER sample, and the remaining (HUNTER) was further labeled with undecanal. Undecanal labelling was performed by adjusting the peptide solution to 40% ethanol, adding 12 μL undecanal (97%), and introducing sodium cyanoborohydride to a final concentration of 30 mM. The mixture was incubated for 1 h at 37°C. The labeled peptides were then acidified to pH 3 using trifluoroacetic acid (TFA), and SepPak tC18 cartridges (1 cc; Waters, Eschborn, Germany) were utilized for undecanal clean‐up. To condition and equilibrate the stationary phase, methanol, 40% ethanol, and 0.1% TFA were used. For binding of undecanal‐labeled internal peptides, samples were loaded onto the cartridges and washed with 0.1% TFA. The eluting fractions (flow‐through) were collected, dried for 3 h in a SpeedVac, and subsequently freeze‐dried overnight. To desalt pre‐HUNTER and HUNTER samples, Pierce C18 Spin Tips (Thermo, Waltham, Massachusetts, USA) and SepPak C18 cartridges (Waters, Eschborn, Germany) were used, respectively. Eluted peptides were vacuum‐dried and kept at −20°C. For the LC–MS analysis, the samples were redissolved in 3% acetonitrile (ACN) containing 0.1% TFA and injected. The Dionex U3000 nanoHPLC system was used for the chromatographic separation. The system was equipped with an Acclaim PepMap100 C18 column (2 μm, 75 μm × 500 mm) that was coupled online to the mass spectrometer. The eluents consisted of eluent A (0.05% formic acid in water) and eluent B (80% ACN with 0.04% formic acid). For pre‐HUNTER samples, separation was completed over a programmed 90‐min run. The following elution parameters were applied: 4% isocratic B for 2 min, followed by an increase from 4% to 50% B over 60 min, 50% to 90% B over 5 min, and a 10‐min hold at 90% B. Inter‐run equilibration was performed at 4% B for 13 min. For HUNTER samples, a 220‐min programmed run was used with 4% B for 2 min, then a linear gradient increase from 4% to 50% B over 180 min, followed by 50% to 90% B over 10 min, and a 13‐min hold at 90% B. Afterwards, a 15 min inter‐run equilibration at 4% B was performed. A constant flow rate of 300 nL/min was maintained, and between each sample injection, wash runs were introduced. A QExactive Plus (Thermo, Waltham, Massachusetts, USA) was used for data acquisition. Full MS spectra were recorded over an m/z range of 350–1400 at a resolution of 70 000. Data‐dependent MS/MS scan acquisition targeted pre‐HUNTER or HUNTER most intense ions (Top 10 or Top 15), using HCD activation at a NCE of 33 and a resolution of 17 500. Dynamic exclusion was set to 40 s. Raw spectral data were processed using Proteome Discoverer 2.2 (Thermo, Waltham, Massachusetts, USA) with the SequestHT algorithm. Searches were conducted against the reviewed *Mus musculus* Uniprot proteome (17 530 entries) [54] supplemented with the common Repository of Adventitious Proteins (cRAP) database. Precursor and fragment ion mass tolerances were set at 10 ppm and 0.02 Da, respectively. Semi‐tryptic digestion with Trypsin_R was specified, permitting up to two missed cleavages. Dynamic modifications included oxidation of methionine (+15.995 Da), N‐terminal acetylation (+42.011 Da), and TMTsixplex addition (+229.163 Da) at the peptide N‐terminus. Static modifications included carbamidomethylation (+57.021 Da) of cysteine, and TMTsixplex modification of lysine was set as a dynamic modification for labeling efficacy calculations. Protein and peptide identifications were filtered using a 1% false discovery rate (FDR) threshold, and strict parsimony principles were applied.

### 2.3 Generation of Meprin α‐Deficient HEK293T Cell Line Using CRISPR/Cas9 Genome Editing

DMEM GlutaMAX medium (Gibco, Waltham, Massachusetts, USA) supplemented with 10% FCS (Gibco, Waltham, Massachusetts, USA) was used to cultivate HEK293T cells. Using CRISPR/Cas9 genome editing, multi‐guide RNA (U*C*U*UUAAAGGUGCUGGUCUG, A*C*A*CGAGAUCCUGCAUGCUU, G*G*G*AUGAUUAUGUGAACAUC) and recombinant *Streptococcus pyogenes* Cas9 2NLS (Synthego, Redwood City, California, USA) induced a deletion in MEP1A. According to Synthego's instructions, Cas9 RNPs were prepared using 100 pmol multi‐guide RNA and 20 pmol Cas9 per electroporation, with 7,6 × 104 cells and the Neon device (Thermo, Waltham, Massachusetts, USA) (2 pulses, 1150 V, 20 ms). To obtain single‐cell clones, single‐cell seeding in 96‐well plates was performed immediately after electroporation. Genomic DNA was extracted from these clones using the GeneJET Genomic DNA Purification Kit (K0721 Thermo, Waltham, Massachusetts, USA). Genotyping by PCRs was performed using the following primers: for: 5′‐CCCCATCTTTGGACCATTAGC‐3′, rev: 5′‐CCATTCCTTGAGGCCCTTATCT‐3′ (Figure S1).

### 2.4 Co‐Transfections

Human embryonic kidney cells (HEK 293 T wt) (a kind gift from M. M. Mueller, RRID:CVCL_0063), with a CRISPR‐Cas9‐mediated meprin β‐knockout (HEK 293 T MEP1B‐knockout [50]), or with a CRISPR‐Cas9‐mediated meprin α‐knockout (HEK 293 T MEP1A‐knockout, generation of the cell line seen above) were cultivated at 37°C and 5% CO_2_ in complete medium, consisting of high‐glucose DMEM (Gibco, Waltham, Massachusetts, USA) with 6 mM glutamine (Gibco, Waltham, Massachusetts, USA), 100 U/mL penicillin (Gibco, Waltham, Massachusetts, USA), 100 U/mL streptomycin (Gibco, Waltham, Massachusetts, USA), and 10% fetal bovine serum/FBS (Gibco, Waltham, Massachusetts, USA). The cells were transfected at approximately 70% confluency. For the co‐transfection, PEI MAX (Polysciences, Warrington, Pennsylvania, USA) was used according to the manufacturer's protocol. Cells were transfected in OptiMEM (Gibco, Waltham, Massachusetts, USA). After 5 h of incubation, the medium was replaced with complete medium. For the co‐transfections, the following plasmids were used: pLHCX (Clontech, Mountainview, USA), pLBCX (Clontech, Mountainview, USA), plBCX‐MEP1B‐HA‐tag (created in our lab [29]), pcDNA4/TO‐MEP1B‐STREP‐tag (a kind gift from C. Becker‐Pauly group), pcDNA4/TO‐MEP1B E153A‐STREP‐tag (a kind gift from C. Becker‐Pauly group), pcDNA4/TO‐MEP1A‐STREP‐tag (a kind gift from the Becker‐Paul group [50]), pAPtag5‐IgK‐NCAN‐full length‐5xHIS‐tag (a kind gift from Cagla Eroglu Addgene_219812 [55]), pCMV6‐Entry‐BCAN‐myc‐flag‐tag (NM_021948 ORIGENE, Rockville, Maryland, USA), which encodes for brevican, and pCMV6‐Entry‐PTPRZ‐myc‐flag‐tag (NM_002851 ORIGENE, Rockville, Maryland, USA), which encodes for RPTPζ. The cells were co‐transfected with a potential meprin β substrate and either human meprin β (MEP1B), or the catalytically inactive human meprin β variant (MEP1B E153A), or human meprin α (MEP1A). pLHCX and pLBCX were used as empty vector controls to ensure consistent DNA and PEI concentrations across all cells. The cells were mechanically detached and washed 24–72 h after transfection (depending on their confluency) with ice‐cold Dulbecco's Phosphate Buffered Saline (Sigma‐Aldrich, St. Louis, Missouri, USA). Cells were lysed with cell lysis buffer [150 mM NaCl, 1% Nonidet P‐40, 50 mM Tris, supplemented with EDTA‐free protease inhibitor cocktail c0mplete (Roche, Basel, Switzerland)]. Protein concentrations in cell lysates and medium were measured using a BCA assay according to the manufacturer's guidelines. All co‐transfections were repeated at least 3 times.

### 2.5 Expression of Fetuin‐B/ Soluble Meprin β, and Activation of Recombinant Soluble Meprin β

The recombinant soluble form of human meprin β (Q16820, T^23^‐K^614^) (sMeprin β) and murine fetuin‐B (Q9QX1) with a C‐terminal pentaHis‐tag were recombinantly expressed in High Five insect cells ( *Trichoplusia ni* ) as previously reported [56, 57, 58]. Recombinant fetuin‐B was expressed in COS‐7 cells as previously described [59]. Fetuin‐B was affinity‐purified on a Ni‐NTA affinity matrix that was equilibrated with 25 mM imidazole, 10 mM Tris–HCl pH 7.4, 200 mM NaCl [58]. To remove unspecifically bound proteins, the imidazole concentration was increased to 50 mM, and for the elution of fetuin‐B, imidazole concentration was again increased to 100 mM [58]. Dialysis or gel filtration was used to remove imidazole, and the purity of fetuin‐B was examined in Coomassie brilliant blue stainings of SDS‐PAGE and western blots [58]. Activation of sMeprin β was achieved using meprin β activation buffer that included bovine trypsin (LS02122, Worthington Biochemical Corp., New Jersey, USA) in a molar ratio of 70:1 in 50 mM Tris, 150 mM NaCl, 0.01% Brij‐35 (pH 7.4) for 45 min at 37°C before addition of cOmplete EDTA‐free protease inhibitor cocktail (final concentration 2.5 mg/mL; Roche, Basel, Switzerland) [56, 58]. Catalytic activity was confirmed via fluorogenic substrate turnover of 25 μM Ac‐RE(Edans)‐DR‐Nle‐VGDDPY‐K(Dabcyl)‐NH2 (Biosyntan, Berlin, Germany) [57, 58].

### 2.6 Brevican Digestion With sMeprin β

For brevican digestion with sMeprin β, HEK 293 T wt cells were transfected with pCMV6‐Entry‐BCAN‐myc‐flag‐tag (NM_021948 ORIGENE, Rockville, Maryland, USA). For cell lysis, EDTA‐free 1× protease inhibitor cocktail (1 mg/mL c0mplete; Roche Basel, Switzerland) was used to prevent sMeprin β impairment in the following digestion. After protein extraction, the cell lysates were incubated for 0, 30, or 60 min at 37°C in a water bath. Lysates were incubated under the following conditions: negative control, meprin β activation buffer molar ratio of 70:1, activated sMeprin β molar ratio of 100:1, activated sMeprin β and fetuin‐B (meprin β inhibitor, a kind gift from Hagen Körschgen [57]) in a molar ratio of 100:1:4, or with fetuin‐B in a molar ratio of 100:4. The reaction was stopped by putting the lysates on dry ice with reducing ROTILoad SDS‐loading buffer (Roth, Karlsruhe, Germany). Digested lysates were used directly afterwards for SDS‐PAGE and western blot analysis.

### 2.7 Cell Surface Biotinylation

To assess if meprin alpha can be located on the cell surface, HEK 293 T wt cells were transiently triple‐transfected with brevican, meprin β, and meprin α. In double‐transfected controls, plHCX served as the empty vector. After transfection, triple‐transfected cells were cultivated for 48 h in complete medium. For the cell surface biotinylation, cells were cooled on ice for 10 min, and the medium was aspirated. Then, cells were washed 3× with ice‐cold PBS‐CM (135 mM NaCl, 2.7 mM KCl, 9.2 mM Na_2_HPO_4_, 1.8 mM KH_2_PO_4_, 0.1 mM CaCl_2_, 1 mM MgCl_2_ pH: 7.4) and incubated with EZ‐Link Sulfo‐NHS‐SS‐Biotin solution (21 331 Thermo, Waltham, Massachusetts, USA; 1 mg/mL in PBS‐CM) for 30 min at 4°C. Afterward, the biotin‐containing solution was aspirated, and cells were incubated with ice‐cold Quenching buffer (PBS‐CM, 50 mM Tris–HCl, pH: 8.0) for 10 min at 4°C. Then, cells were washed three times in ice‐cold PBS‐CM to remove any unbound biotin and quenching buffer. Cells were mechanically detached in PBS‐CM and lysed with cell lysis buffer [150 mM NaCl, 1% Nonidet P‐40, 50 mM Tris, supplemented with EDTA‐free protease inhibitor cocktail c0mplete (Roche, Basel, Switzerland)]. For the pulldown of biotinylated protein, the protein concentration in the cell lysates was determined by BCA assay to ensure equal protein loading. Pierce Neutravidin Agarose beads (29 201 Thermo, Waltham, Massachusetts, USA) were equilibrated according to the manufacturer's protocol, and incubated with cell lysates overnight at 4°C on a rotating wheel. The next day, beads were washed three times in cell lysis buffer, then boiled at 95°C in ROTILoad SDS‐loading buffer (Roth, Karlsruhe, Germany) for SDS‐PAGE and western blot analysis.

### 2.8 SDS‐PAGE and Western Blotting

Protein extracts of cell lysates or medium were separated using SDS‐PAGE. 20 μg of protein per well was loaded for the analysis. The separated protein extracts were transferred to 0.45 μm nitrocellulose membranes (Amersham Hybond ECL) and blocked with 5% milk powder in Tris‐buffered saline with Tween 20 [137 mM NaCl, 2.7 mM KCl, 25 mM Tris Base, 0.1% Tween 20]. Protein extractions were analyzed for different ECM proteins using the following antibodies: rabbit‐anti‐MYC‐tag (2278 Cell Signaling technology, Denvers, Massachusetts, USA, RRID:AB_490778), mouse‐anti‐STREP‐tag (34 850 Qiagen, Hilden, Germany, RRID:AB_2810987), rat‐anti‐HA‐tag 3F10 (11 867 427 001 Roche, Basel, Switzerland), mouse‐anti‐Flag‐tag (TA50011 ORIGENE, Rockville, Maryland, USA, RRID:AB_2622345), rabbit‐anti‐6xHis‐tag (GTX115045 Genetex, Irvine, California, USA, RRID:AB_2036130), mouse‐anti‐α‐tubulin (62 204 Invitrogen, Carlsbad, California, USA), rabbit‐anti‐actin (A5060 Sigma‐Aldrich, St. Louis, Missouri, USA, RRID:AB_476738), and rabbit‐anti‐brevican (19017–1‐AP ptglab, Rosemont, Illinois, USA, RRID:AB_10643526). The following dye‐coupled secondary antibodies were used, depending on the species of the primary antibody: IRDye800CW and IRDye 680RD goat‐anti‐mouse, goat‐anti‐rat, and ‐rabbit (D50805‐03, 926–32 211, 926–32 211, D10217‐05, 926–68 071 Licor Biotech, Lincoln, Nebraska, USA, RRID:AB_621843, AB_621843, AB_10956166), and StarBright Blue 700 and StarBright Blue 520 goat‐anti‐mouse and ‐rabbit (12 005 866, 12 005 869, 12 004 158, 12 004 161 BioRad Laboratories, Hercules, California, USA, RRID:AB_2934034, AB_2884949, AB_3712167, AB_2721073). Membranes were developed on a ChemiDoc MP (BioRad Laboratories, Hercules, California, USA). Protein expression was normalized to the respective loading control (α‐tubulin or actin).

### 2.9 Transmission Electron Microscopy

One mepβ^Cre;wt/wt^ and one mepβ^Cre;TG/wt^ mouse were transcardially perfused with a fixing solution consisting of 2.5% glutaraldehyde and 2% paraformaldehyde in 0.1 M phosphate buffer, pH 7.4. Brains were then isolated and post‐fixed overnight at 4°C in fixing solution. The brains were washed three times in 0.1 M phosphate buffer for 5 min and cut at a thickness of 200 μm with a vibratome (Leica VT1200, Leica Microsystems, Nussloch, Germany). Sections containing the dorsal hippocampus (Bregma between −1.43 mm and −1.91 mm) were selected, and the CA1 layer was dissected under a stereomicroscope (Leica M80, Leica Microsystems, Nussloch, Germany) and stored overnight at 4°C in 0.1 M phosphate buffer. The CA1 sections were then post‐fixed in 2% OsO_4_ for 90 min at room temperature. After washing twice in 0.1 M phosphate buffer for 5 min, the specimens were incubated twice for 15 min in 30% and 50% ethanol solutions. They were left overnight in 70% ethanol. The following day, they were incubated twice for 15 min in 90% ethanol and four times for 15 min in 100% ethanol. For embedding in Epon, the specimens were incubated twice for 10 min in propylene oxide. They were then incubated in an Epon‐propylene oxide solution: first for 30 min (1:2 ratio), then for 2 h (1:1 ratio), and finally for 2 h (2:1 ratio), before incubation in pure Epon overnight. The pure Epon was then polymerized in an oven for 2 days at 65°C. The blocks were trimmed and cut with an ultramicrotome (Reichert‐Jung, Ultracut E) at 70 nm. Some semithin sections were stained according to Richardson [60] in order to confirm proper localization in the CA1, stratum radiatum. The slices were mounted on 3.05 mm copper TEM support grids (Agar scientifics). Post‐contrasting was performed by incubating the slices with 4% uranyl‐acetate for 8 min at 55°C in the dark, followed by washing in water, drying for 30 min on filter paper, and then staining with lead citrate according to Reynolds [61] for 2 min. The slices were finally washed in water and dried. Randomly selected sections were imaged at a 21 560× magnification with a LEO 906 transmission electron microscope (Zeiss, Oberkochen, Germany). A total of 25 images for the mepβ^Cre;wt/wt^ mouse and 22 images for the mepβ^Cre;TG/wt^ mouse were analyzed. The density of synapses was calculated in a blinded manner using the software ImageSP as the ratio of the total number of synapses to the surface area of each image. The results are expressed as a percentage of mepβ^Cre;wt/wt^.

### 2.10 Immunofluorescence Staining

4% paraformaldehyde (PFA) was used to perfuse brains from 10‐month‐old mice (*n* = 3 per genotype). Brains were immersed in 20% sucrose, then 30% sucrose, until fully submerged at the bottom of a 15 mL Falcon tube. Then the brains were frozen on dry ice. When completely frozen, the brains were embedded in tissue‐freezing medium (Leica Microsystems, Nussloch, Germany) and cut into 50 μm sagittal sections using a CM3050S cryostat (Leica Microsystems, Nussloch, Germany). Afterwards, sections were transferred into cryoprotective solution (25% polyethylene glycol, 25% glycerol, and 50% PBS), and stored at 4°C. For Immunofluorescence staining, the sections were processed as free‐floating slices and washed with PBS. They were blocked and slightly permeabilized by incubation in blocking solution containing 0.5% Triton X‐100 and 5% bovine serum albumin (BSA) (A9418 Sigma‐Aldrich, St. Louis, Missouri, USA) in PBS for 3 h at room temperature. Afterwards, the sections were incubated with biotinylated Wisteria floribunda Agglutinin (WFA) (B‐1355‐2 Vector Laboratories, Newark, California, USA) to stain for PNNs at 4°C overnight in 2,5% BSA blocking solution. Sections were washed with PBS and incubated with Alexa Fluor 488‐conjugated Streptavidin (S11223 Invitrogen, Carlsbad, California, USA) for 3 h at room temperature. Then sections were washed in PBS and incubated with 4′, 6‐diamidino‐2‐phenylindole (DAPI) (62 248 Thermo, Waltham, Massachusetts, USA) in PBS for 10 min. Finally, the sections were rinsed and mounted using Fluoromount‐G (00–4958‐02 Thermo, Waltham, Massachusetts, USA). Stained sections were stored at 4°C until Confocal Imaging.

### 2.11 Confocal Imaging of the Hippocampal CA1 Region

WFA‐stained sagittal brain slices were imaged with a Zeiss LSM 710 confocal laser scanning microscope (Zeiss, Oberkochen, Germany). First, the hippocampal CA1 region was imaged using a 25× magnification. WFA‐stained PNNs were always imaged in 12 steps through the z‐plane at 100× magnification. To detect the fluorophores, the following acquisitions were used: WFA (Alexa Fluor 488, pseudo green) exited at 488 nm with a pinhole size of 79.9 μm and a gain of 1100, and DAPI (pseudo blue) exited at 405 nm with a pinhole size of 64.6 μm and a gain of 1200. Pictures were generated at a frame size of 1280 × 1280 pixels, with line averaging set to 16 and a bit depth of 12. Subsequently, images were processed in ImageJ by splitting and merging channels and adding a 20 μm scale bar for 100× magnification and a 100 μm scale bar for the 25× magnification. All settings were kept identical for the sections of mepβ^Cre;TG/wt^ and mepβ^Cre;wt/wt^ mice.

### 2.12 Quantification and Statistical Analysis

Protein expression on western blots was analyzed using densitometric Quantification in ImageJ v.1.52(NIH) [50]. All Graphs were created in GraphPad Prism 8 software (LA Jolla). For all groups, the standard error of the means (SEM) and means were calculated and presented graphically. For the densitometric quantification of western blots and assessment of statistical significance, loading control normalized raw protein levels from each blot were paired and compared using the paired *t*‐test (for 2 groups), and the RM one‐way ANOVA followed by Tukey's multiple comparison test (for > 2 groups). The significance levels were displayed as ns: *p* > 0.05; *p ≤ 0.05; ** *p* ≤ 0.01; ***p ≤ 0.001; ****p ≤ 0.0001. For statistical analysis, only technical replicates of *n* = 3 or more were used. Figure Descriptions were used to specify the respective statistical tests used. Microsoft PowerPoint 365 v.2304, BioRender.com (includes BioRender contents licensed under the OpenAccess Publication license), and GraphPad Prism 8 v.8.02 were used to create the Figures.

## 3 Results

### 3.1 Meprin β Cleaves Brevican at Amino Acid Positions E461_↓_ E462, K464_↓_ E465, and E584_↓_ T585

**FIGURE 1:**
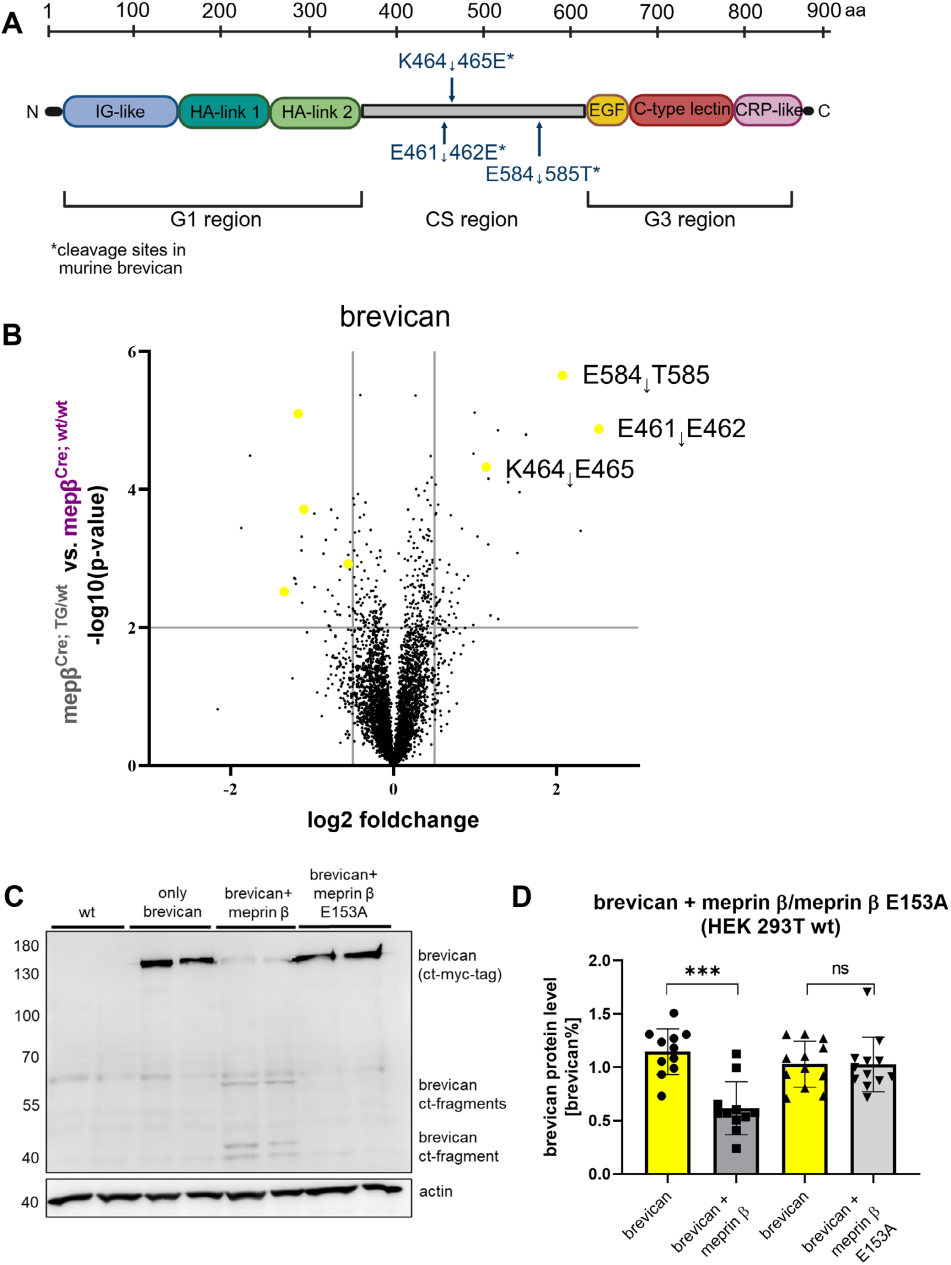
Meprin β cleaves brevican at amino acid positions E461_↓_E462, K464_↓_E465, and E584_↓_T585. (A) Schematic illustration of brevican protein structure with meprin β cleavage sites identified by the N‐terminomics analysis of murine brevican [50]. Brevican consists of a N‐terminal G1 domain with an immunoglobulin‐like loop (IG‐like), two hyaluronic acid link domains (HA‐link 1, 2), a chondroitin sulfate (CS) attachment region, and a C‐terminal G3 domain with an epidermal growth factor (EGF)‐like domain, a c‐type lectin‐like domain, and a complement regulatory protein (CRP)‐like domain. Cleavage sites are in the CS attachment region, where CS chains physiologically attach to brevican. (B) Volcano plot of the N‐terminomics results. mepβ^Cre;TG/wt^ were compared to mepβ^Cre;wt/wt^ animal brain lysates. Underrepresented N‐terminal brevican fragments in mepβ^Cre;TG/wt^ are shown on the left, and overrepresented brevican fragments on the right, both highlighted in yellow. A threshold of log_2_ (difference) = ±0.5 and ‐log10 (p‐value) = 2 was applied; *n* = 3. Cleavage sites of newly identified N‐terminal fragments are indicated by P1_↓_P1′ amino acid residues: E461_↓_E462, K464_↓_E465, And E584_↓_T585. (C) Representative western blot of co‐transfections in HEK 293 T wt cells. 20 μg of protein of solo brevican transfected, brevican + meprin β co‐transfected, and brevican + meprin β E153A (inactive variant) co‐transfected cell lysates were loaded, with wildtype cell lysates as a negative control. For loading control brevican was normalized to Actin. Brevican was detected, using an anti‐Myc tag antibody. (D) Densitometric quantification and statistical analysis of (C) was performed using the paired *t*‐test. Significance levels were indicated as ns: *p* > 0.05; *p ≤ 0.05; **p ≤ 0.01; ***p ≤ 0.001 (*n* = 8).

To determine meprin β cleavage in brevican, N‐terminomics analysis was performed on brain lysates from 12‐month‐old mepβ^Cre;wt/wt^ and mepβ^Cre;TG/wt^ animals [50]. This analysis revealed a significant increase of three N‐terminal brevican fragments in transgenic mice. All three fragments result from acidic cleavage sites (E in P1/P1′) matching the typical meprin β cleavage specificity (Figure 1B). The fragment closest to the N‐terminus of the protein, with a calculated molecular weight of ~46 kDa, is cleaved between E461_↓_E462. The second closest to the N‐terminus is generated by cleavage between K464_↓_E465, leaving a fragment that is only a few amino acids smaller. The third closest to the N‐terminus is cleaved between E584_↓_T585 (Figure 1B). Interestingly, N‐terminomics revealed four brevican fragments that were significantly underrepresented in the brains of mepβ^Cre;TG/wt^ mice (Figure S2). The fragment closest to the N‐terminus of the protein is cleaved directly behind the signal peptide at A22_↓_D23. The fragment second closest to the N‐terminus is cleaved at S406_↓_E407, while the fragments third and fourth closest to the N‐terminus are cleaved only a few amino acids downstream at D408_↓_G409 and G412_↓_S413 (Figure 1B). These fragments did not fit any previously described brevican cleavage sites for other proteases [62]. However, this indicated inhibition of proteolytic processes other than meprin β.

To further validate the proteolytic cleavage of human brevican by meprin β, myc‐tagged human brevican was transiently co‐transfected with either human meprin β or the catalytically inactive human meprin β E153A variant (Figure 1C). SDS Page and western blot analysis of cell lysates revealed a significant decrease in full‐length brevican in the meprin β‐, but not in meprin β E153A‐transfected cells. These results confirm proteolysis of fully matured human brevican by human meprin β in vitro (Figure 1C,D).

### 3.2 Soluble Recombinant Meprin β Homodimers Cleave Brevican

**FIGURE 2:**
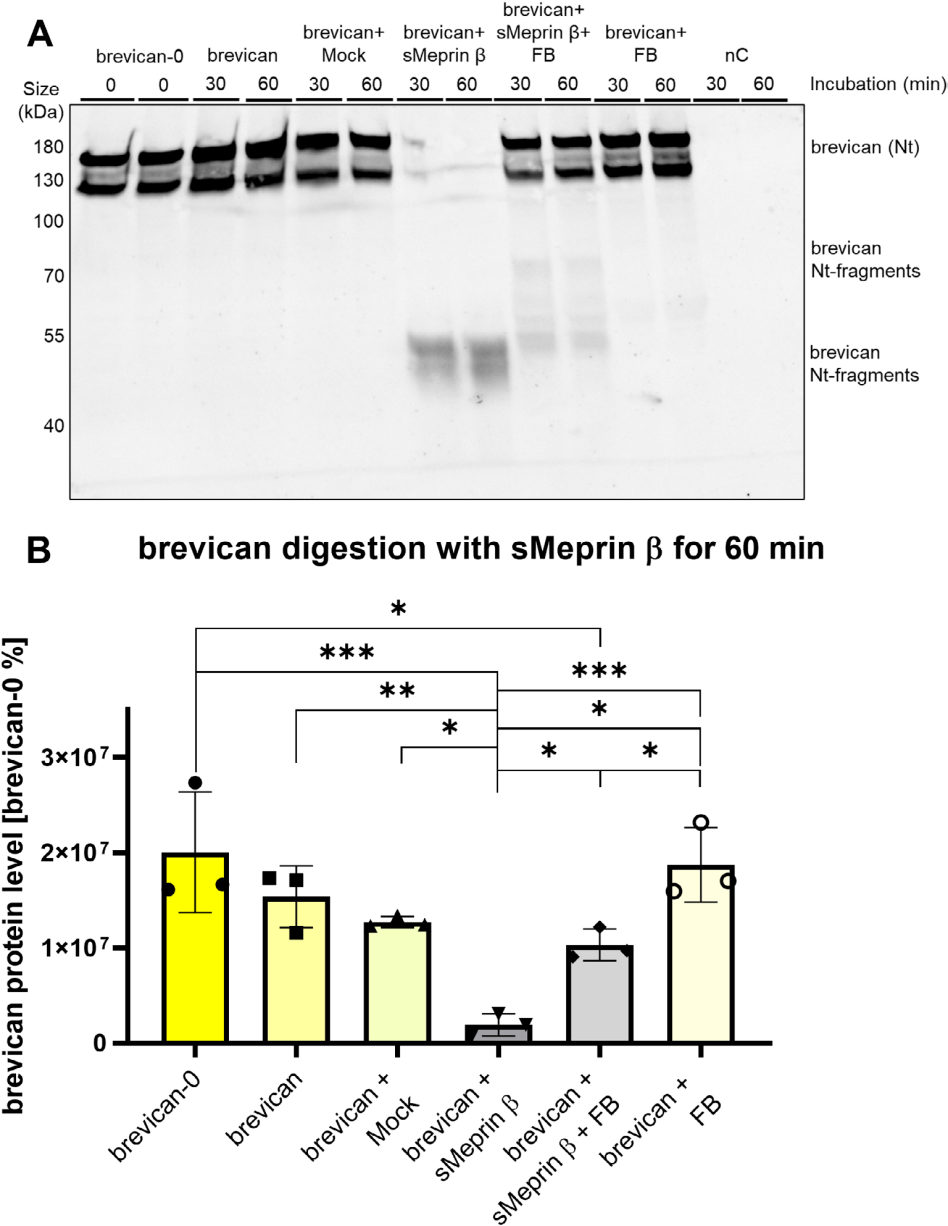
Brevican cleavage by soluble recombinant meprin β homodimers. (A) Representative western blot loaded with 20 μg protein of brevican‐transfected HEK 293 T cell lysates that were incubated for 0, 30, or 60 min either alone: Brevican, or with mock solution: Brevican+mock (containing meprin β activation buffer, molar ratio of 70:1), or with activated recombinant soluble meprin β (sMeprin β): Brevican+sMeprin β (molar ratio of 100:1), or with activated sMeprin β and fetuin‐B (meprin β inhibitor, molar ratio of 100:1:4): Brevican+sMeprin β + FB, or just with fetuin‐B (molar ratio of 100:4): Brevican+FB. Brevican was detected with an N‐terminal polyclonal antibody. (B) Densitometric quantification and statistical analysis of 60 min incubation time were performed using RM one‐way ANOVA, and all column means were compared using Tukey's multiple comparisons test. Significance levels were indicated as ns: *p* > 0.05; *p ≤ 0.05; **p ≤ 0.01; ***p ≤ 0.001 (*n* = 3).

To test whether shed recombinant meprin β homodimers (sMeprin β) can digest full‐length brevican, HEK 293 T cells were transfected with brevican, lysed, and incubated for either 0/30/60 min alone, with mock solution (including meprin β activation buffer with trypsin in a molar ratio of 70:1), with activated and recombinant sMeprin β (molar ratio of 100:1), with activated sMeprin β and fetuin‐B (meprin β inhibitor, molar ratio of 100:1:4), or just with fetuin‐B (molar ratio of 100:4) (Figure 2A). The full‐length brevican protein was detected with an anti‐brevican antibody that targets its N‐terminus. A significant decrease in full‐length brevican was observed in cell lysates incubated with activated sMeprin β when compared to 60 min incubated brevican with mock solution. This decrease could be partially rescued by the addition of fetuin‐B. Incubating cell lysates for 30 or 60 min, either alone or with the mock solution, did not alter full‐length brevican protein levels (Figure 2B). This in vitro analysis suggested that sMeprin β can cleave full‐length brevican and confirmed brevican as a direct meprin β substrate. Moreover, the N‐terminal antibody detected two bands: the larger one, at approximately 145 kDa, that most likely represents the full‐length protein, and a smaller one that could either be the deglycosylated protein or the alternatively spliced GPI‐anchored variant of brevican [63]. Interestingly, our data suggest that both full‐length variants can be cleaved by meprin β.

### 3.3 Brevican Cleavage by Meprin β Homodimers With Meprin α Co‐Expression

**FIGURE 3:**
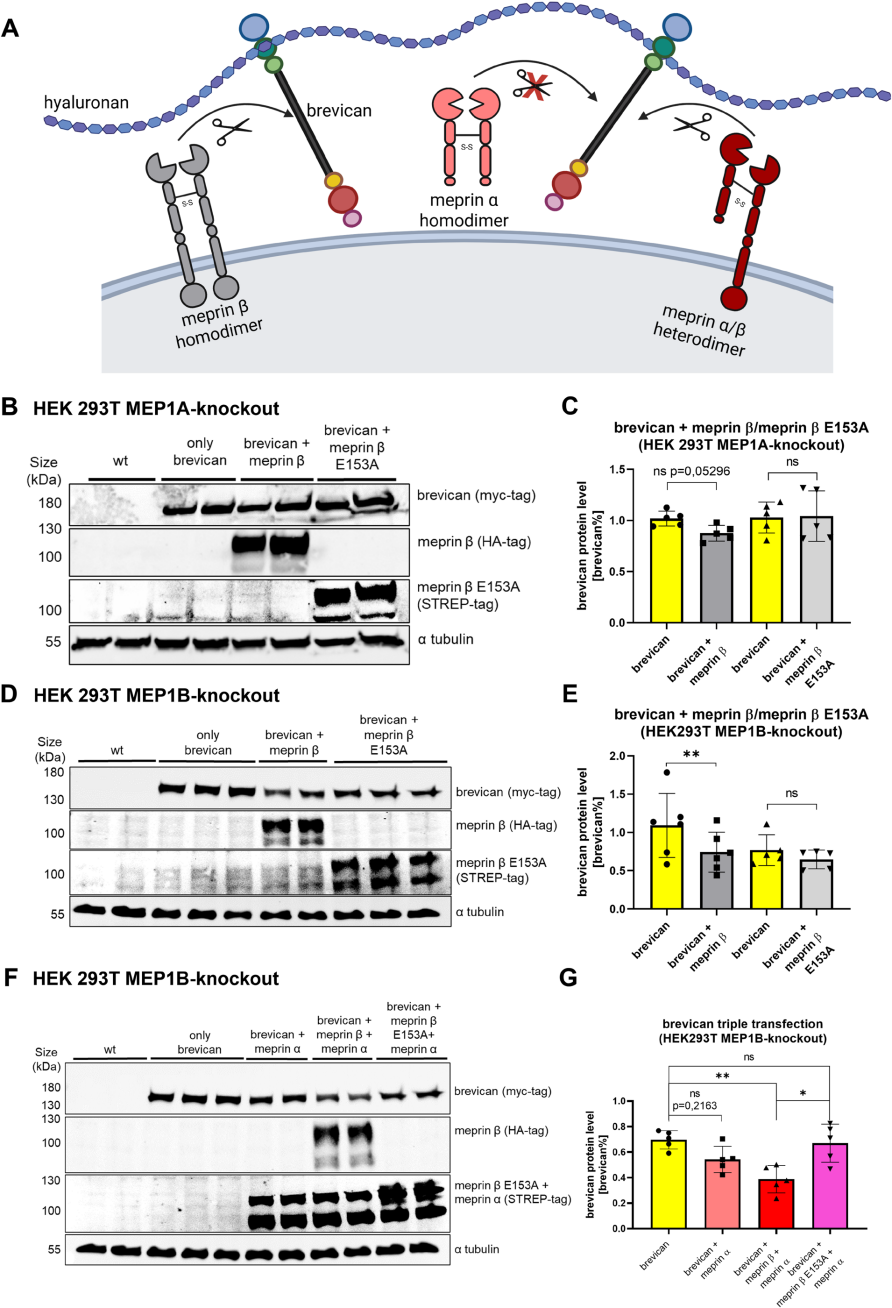
Brevican cleavage by meprin β with meprin α co‐expression. (A) Schematic illustration of brevican cleavage by meprin β homodimers and meprin α/β heterodimers. (B) Representative western blot of co‐transfections in HEK 293 MEP1A‐knockout cells. 20 μg protein of wt, brevican, brevican + meprin β, and brevican + meprin β E153A transfected cells were loaded. (C) Densitometric quantification of these western blots. The graphs show the arithmetic means ± SD of 5 replicates from five independent experiments (*n* = 5). Statistical analysis was performed using the paired *t*‐test. (D) Representative western blot of brevican and meprin β or meprin β E153A co‐transfections in HEK293T MEP1B‐knockout cells. 20 μg protein of wt, brevican, brevican + meprin β, and brevican + meprin β E153A transfected cells were loaded. (E) Densitometric quantification and statistical analysis with the paired t‐test. Graphs show the arithmetic means ± SD of 6 replicates from five independent experiments (*n* = 5) for meprin β and 5 replicates from 3 independent experiments for meprin β E153A (*n* = 3). (F) Representative western blot of triple co‐transfections of brevican, meprin β, meprin β E153A, and meprin α. 20 μg protein of brevican, brevican + meprin α, brevican + meprin β + meprin α, and brevican + meprin β E153A + meprin α transfected cells were loaded. (G) Densitometric quantification of western blots was performed using RM one‐way ANOVA with Tukey's multiple comparison test. The graph shows the arithmetic means ±SD of 5 replicates from three independent experiments. All significance levels were indicated as ns: *p* > 0.05; *p ≤ 0.05; **p ≤ 0.01; ***p ≤ 0.001.

In meprin α/β heterodimers, the meprin α and meprin β subunits have been shown to exhibit enhanced catalytic activity in a fluorogenic activity assay [49]. Therefore, we investigated the role of meprin α in the cleavage of brevican. For this, HEK293T cells with a CRISPR‐Cas9‐mediated meprin α‐knockout (HEK 293 T MEP1A‐knockout) were co‐transfected with brevican and meprin β. In these cells, no significant reduction of full‐length brevican was detected in co‐transfection with meprin β compared to solo brevican‐transfected cells (Figure 3B,C). However, there was a non‐significant trend of lower full‐length brevican levels in co‐transfections with meprin β (*p* = 0.05296). We could also not observe a reduction of full‐length brevican in co‐transfections of brevican with the catalytically inactive variant of meprin β E153A. These findings suggest that meprin α may contribute to efficient cleavage of brevican.

To test if meprin α alone is also capable of cleaving brevican, firstly brevican together with meprin β or meprin β E153A were co‐transfected into HEK 293 T cells with a CRISPR‐Cas9‐mediated meprin β‐knockout (HEK 293 T MEP1B‐knockout [50]). In these co‐transfections, results appeared similar to those in HEK293T wt cells. A significant reduction in full‐length brevican was only observed in cells co‐transfected with brevican and meprin β, and not observed in cells co‐transfected with brevican and meprin β E153A (Figure 3D,E). This again suggests that meprin β contributes substantially to brevican cleavage, and that meprin α may play a crucial role in it. Therefore, brevican cleavage by meprin α homodimers alone in HEK293T MEP1B‐knockout cells was investigated by co‐transfection with brevican and meprin α. Due to the lack of meprin β in these cells, there could be no heterodimerization of meprin α in double transfections with brevican, resulting only in meprin α homodimers. Although there was a trend of reduced brevican level in co‐transfections with meprin α, our data suggest that brevican was not substantially cleaved by meprin α homodimers, as no significant reduction in full‐length brevican protein level was observed in co‐transfected cell lysates compared with those from cells transfected only with brevican (Figure 3G; *p* = 0.2163). Moreover, triple co‐transfections of HEK 293 T MEP1B‐knockout cells with brevican, meprin β, and meprin α resulted in significantly lower brevican protein levels compared to only brevican‐transfected cells (Figure 3G). This indicates that co‐expression of meprin β and meprin α enhances meprin β‐mediated brevican processing when compared with the expression of either protease alone. However, it remains unclear if this effect is mediated by meprin α/β heterodimerization, altered cellular localization, or changes in protease activity.

To further explore if meprin α contributes directly to brevican processing in co‐expression with catalytically inactive meprin β E153A, HEK293T MEP1B‐knockout cells were co‐transfected with brevican, meprin α, and meprin β E153A. No significant difference in full‐length brevican between solo‐brevican and brevican, meprin α, meprin β E153A co‐transfected cells was observed. Under these experimental conditions, we could not detect a significant contribution of meprin α subunits to brevican cleavage when co‐expressed with catalytically inactive meprin β (Figure 2H,I).

In summary, our data suggest a central role of meprin β in brevican cleavage. However, meprin α seems to play an important role in the cleavage of brevican by possibly enhancing meprin β's catalytic activity when co‐expressed. Nevertheless, the present experiments do not allow us to determine whether this effect results from meprin α/β heterodimerization or effects on meprin β localization, stability, or activity. Interestingly, we found no evidence of substantial brevican cleavage by meprin α alone, neither as a homodimer nor when co‐expressed with catalytically inert meprin β E153A.

### 3.4 Neurocan Cleavage in N‐Terminomics

**FIGURE 4:**
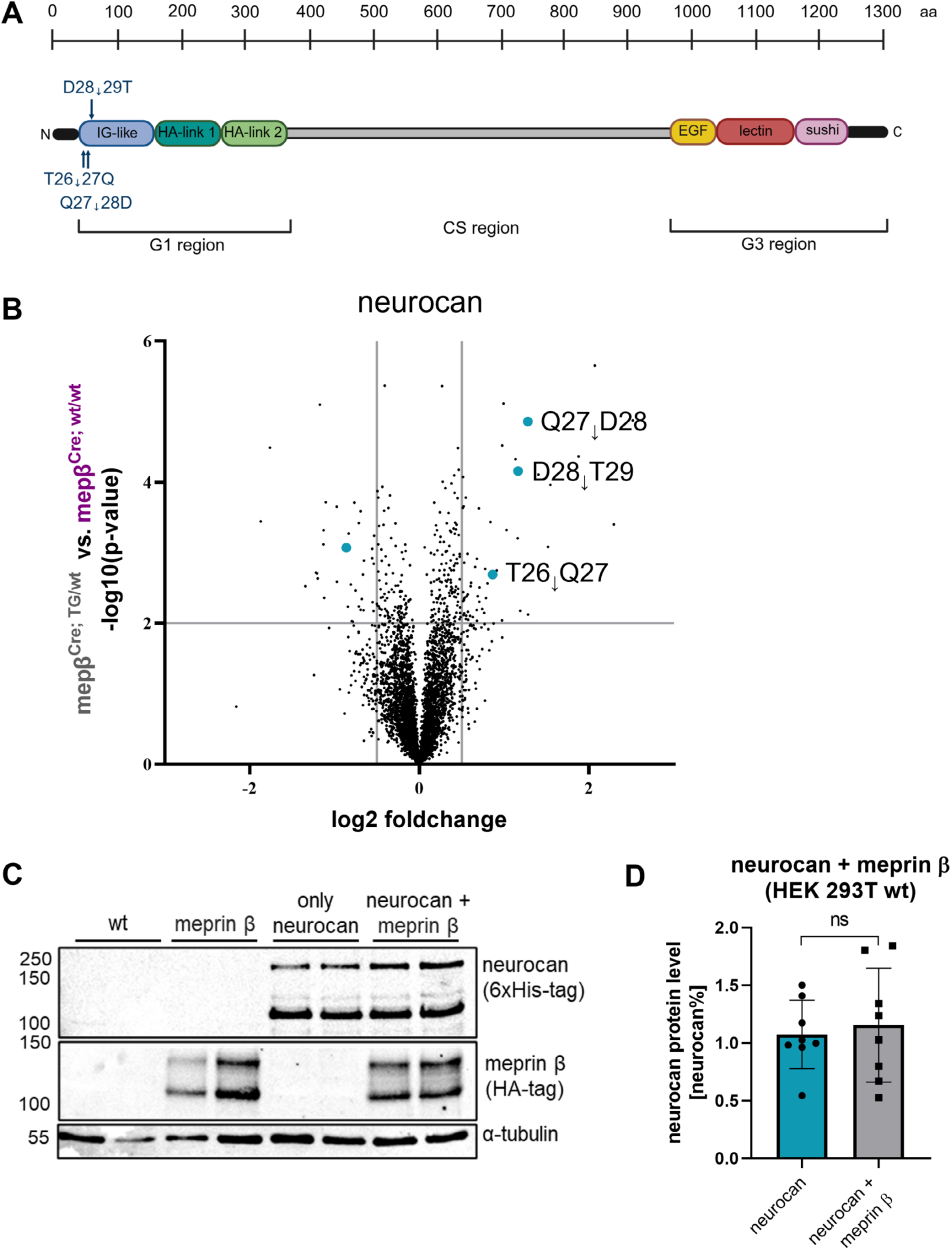
Potential neurocan cleavage in HUNTER N‐terminomics could not be replicated in vitro. (A) Schematic neurocan protein structure with meprin β cleavage sites. Like brevican, neurocan consists of an N‐terminal G1 region with an IG‐like domain, two HA‐link (1, 2) domains, a CS attachment region, and a G3 region with an EGF‐like domain, a lectin‐like domain, and a complement control protein (sushi) region. The cleavage sites are located in the IG‐like domain of neurocan, in close proximity to the signal peptidase cleavage site and are indicated as P1_↓_P1′. (B) Volcano plot of the HUNTER N‐terminomics results comparing mepβ^Cre;TG/wt^ to mepβ^Cre;wt/wt^ animals. A threshold of log_2_ (difference) = ±0.5 and −log10 (*p*‐value) = 2 was applied. Underrepresented N‐terminal neurocan fragments on the left and overrepresented neurocan fragments on the right, both highlighted in turquoise. Cleavage sites of neurocan between T26_↓_Q27, Q27_↓_D28 and D28_↓_T29. Cleavage sites are conserved in human neurocan except D28_↓_T29, which corresponds to D28_↓_I29. (C, D) Western blot analysis of 30 μg protein of neurocan and meprin β co‐transfected HEK293T cell lysates: Solo neurocan, neurocan + meprin β, and wildtype cell lysates for negative control. Graphs show the arithmetic mean ± SD of 8 replicates from five independent experiments (*n* = 5). Densitometric quantification and statistical analysis with the paired *t*‐test with significance levels indicated as ns: *p* > 0.05; *p ≤ 0.05; **p ≤ 0.01; ***p ≤ 0.001.

Apart from brevican, neurocan was identified in the N‐terminomics analysis as a new probable meprin β substrate. Neurocan is another CSPG, part of the lectican family, and a structural component of PNNs [15]. In the N‐terminomics, three significantly overrepresented N‐terminal peptides were found in mepβ^Cre;TG/wt^ animals compared to mepβ^Cre;wt/wt^ animals. All these peptides feature an N‐terminus near the signal peptidase cleavage site (G22_↓_E23 [54]). The peptide closest to the N‐terminus is generated by cleavage at T26_↓_Q27, the second closest to the N‐terminus is cleaved at Q27_↓_D28, and the third closest to the N‐terminus is cleaved directly downstream at D28_↓_T29 (Figure 4B). One fragment, generated by cleavage at R709_↓_S710, was underrepresented in mepβ^Cre;TG/wt^ animals compared to mepβ^Cre;wt/wt^ animals. Neurocan cleavage at this site is not known. However, these results indicate proteolytic cleavage by a protease other than meprin β.

To further investigate the potential cleavage of neurocan by meprin β, we co‐transfected C‐terminally 6xHIS‐tagged neurocan and meprin β into HEK 293 T cells. No significant changes were detected for full‐length neurocan protein levels (Figure 4C,D). However, it must be acknowledged that a small difference of 6 amino acids as expected from the N‐terminomics results cannot be resolved in SDS‐PAGE of > 150 kDa protein lengths with a C‐terminal 6xHIS‐tag.

Therefore, our SDS‐PAGE and western blot results did not allow reliable detection of neurocan cleavage using the current assay configurations. Thus, no definitive conclusion regarding meprin‐mediated processing of neurocan can be drawn, although according to the N‐terminomics results the N‐terminal cleavage is likely minimal.

### 3.5 RPTPζ Is Cleaved by Meprin β and Meprin α

**FIGURE 5:**
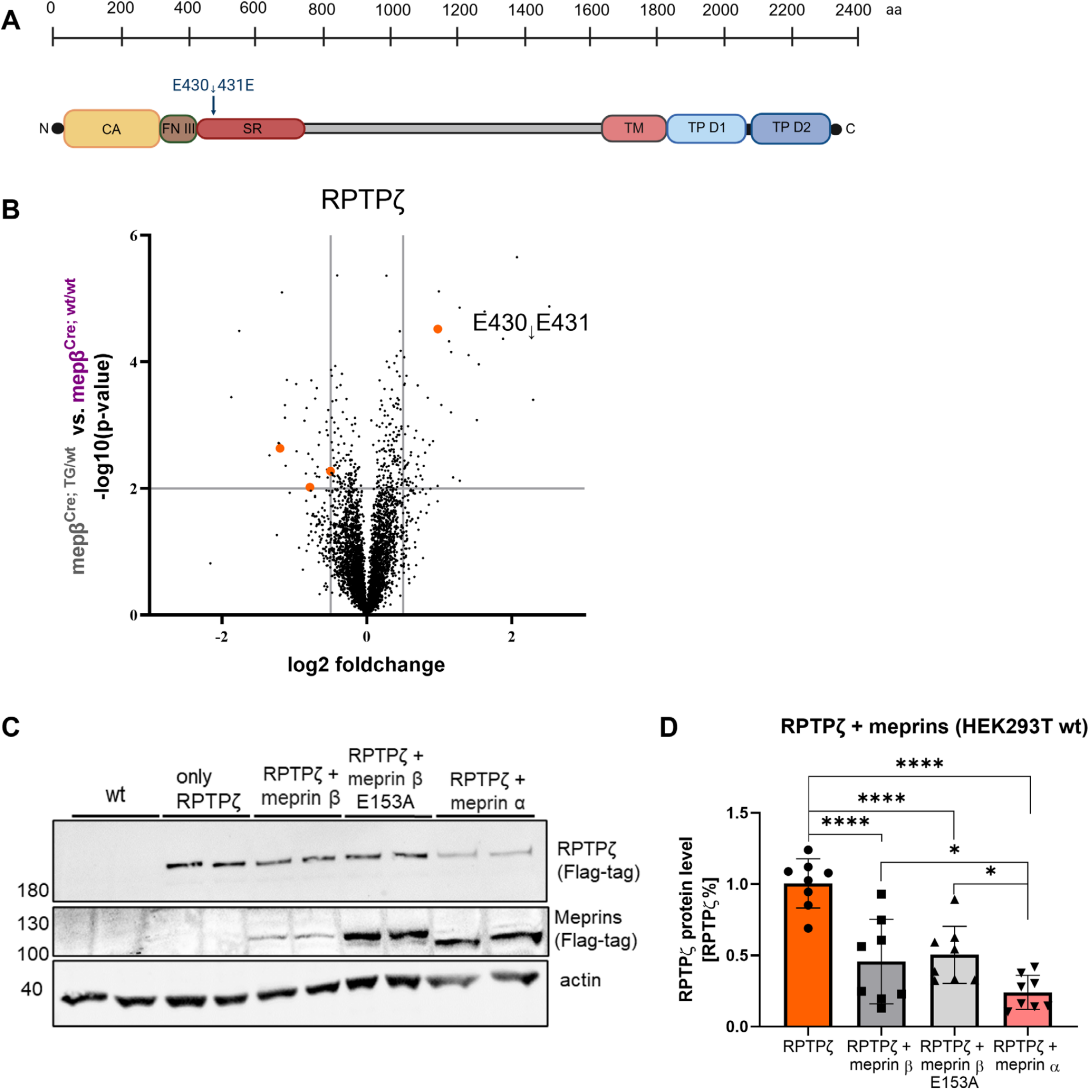
RPTPζ is cleaved by meprin β and meprin α. (A) Schematic protein structure of RPTPζ with meprin β cleavage sites. RPTPζ consists of a carboanhydrase region (CA), a fibronectin III region (FNIII), a spacer region (SR), a chondroitin sulfate binding region, a transmembrane domain (TM), and two tyrosine phosphatase domains (TP D1, TP D2). The cleavage site is located in its spacer region (B) Volcano plot of the HUNTER N‐terminomics analysis results. Here, RPTPζ was identified as a potential substrate of meprin β when comparing mepβ^Cre;TG/wt^ to mepβ^Cre;wt/wt^ animals when a threshold of log_2_ (difference) = ±0.5 and −log10 (*p*‐value) = 2 was applied. One N‐terminal fragment, generated by cleavage between E430_↓_E431, was significantly overrepresented in mepβ^Cre;TG/wt^ animals. This cleavage site is conserved in human RPTPζ. (C) Representative western blot of 30 μg protein of RPTPζ co‐transfections in HEK 293 T wt cells. Cells were either transfected with RPTPζ alone, with RPTPζ and meprin β, with RPTPζ and the catalytically inactive variant meprin β E153A, or with RPTPζ and meprin α. (D) Quantification and statistical analysis was performed with RM one‐way ANOVA, and all column means were compared using Tukey's multiple comparison test. Graphs show the arithmetic mean ± SD of 8 replicates from five independent experiments (*n* = 5). Significance levels were indicated as ns: *p* > 0.05; *p ≤ 0.05; **p ≤ 0.01; ***p ≤ 0.001; ****p ≤ 0.0001.

RPTPζ is a membrane‐bound receptor involved in synaptic plasticity and learning [64, 65]. It is a critical part of PNN formation by anchoring HA chains to the cell in cooperation with tenascin‐R [65]. Furthermore, RPTPζ exists as an alternatively spliced variant called phosphacan that lacks its C‐terminal part TM, TP D1, and TP D2 region. Phosphacan is a key structural component of PNNs as well [66]. RPTPζ was identified in the N‐terminomics analysis as a probable meprin β substrate. In mepβ^Cre;TG/wt^ animals, one fragment, generated by cleavage between amino acids E430_↓_E431, was significantly overrepresented when compared to mepβ^Cre;wt/wt^ animals. This cleavage site E430_↓_E431 closely matches both meprin α's and meprin β's preference for negatively charged amino acids (Figure 5A,B).

To confirm RPTPζ cleavage by meprin β and to investigate the role of meprin α on its proteolysis, RPTPζ was co‐transfected with either meprin β, meprin β E153A, or meprin α in HEK 293 T wt cells. RPTPζ full‐length protein levels were significantly decreased in cells co‐transfected with meprin β, meprin β E153A, or meprin α (Figure 5C,D). This suggests that RPTPζ can be cleaved by meprin α and meprin β subunits. Interestingly, the full‐length RPTPζ protein level is decreased in the presence of meprin β E153A as well. Given that meprin β E153A is a catalytically inert variant, and that our data suggest that meprin α subunits may cleave RPTPζ, one possible explanation might be that meprin α subunits that heterodimerize with meprin β E153A subunits cleaved RPTPζ (Figure 5D).

### 3.6 Meprin β Mediated Effects on PNN Structure and Synapse Density in mepβ^Cre;TG/wt^ Mice

**FIGURE 6:**
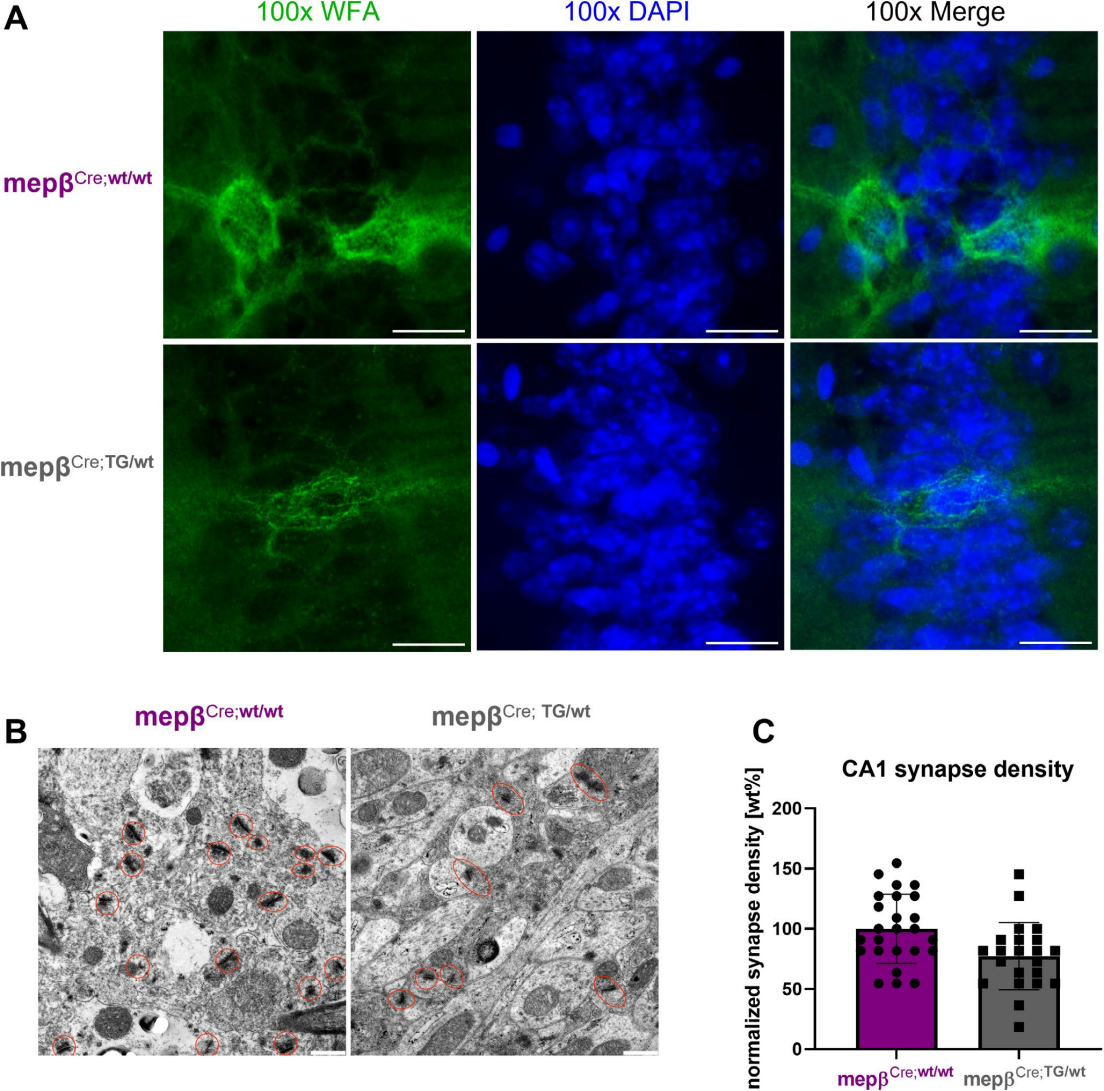
Meprin β mediated effects on PNN structure and synapse density in mepβ^Cre;TG/wt^ mice (A) Representative indirect immunofluorescence images of the hippocampal CA1 region were stained using biotinylated WFA and Streptavidin‐AF 488. PNNs appear to be less dense and overall structurally impaired in mepβ^Cre;TG/wt^ mice compared to their wild‐type counterparts. (B) Representative EM images showing synapses (encircled in red) in the hippocampal CA1 region. (C) Quantification of synapse density in the hippocampal CA1 region comparing mepβ^Cre;TG/wt^ to mepβ^Cre;wt/wt^ mice (*n* = 1). Due to a lack of replicates, no statistical analysis was performed.

The PNN structure is described as a lattice‐like net comprised of CSPGs, linking peptides such as tenascins and HAPLN1, and hyaluronic acid [5]. To test whether the proteolytic processing of brevican and RPTPζ influences PNN structure, brain slices of mepβ^Cre;TG/wt^ and mepβ^Cre;wt/wt^ mice were stained with biotinylated *Wisteria floribunda* Agglutinin (WFA), a commonly used marker for PNNs. For imaging, the hippocampal CA1 region was chosen, as a previous study reported impaired CA1 long‐term potentiation and hippocampus‐based impairment of spatial memory acquisition in mepβ^Cre;TG/wt^ animals [50]. In mepβ^Cre;wt/wt^ animals, PNNs appeared as dense nets surrounding neurons, with small holes that leave space for tightly enwrapped synapses. Interestingly, mepβ^Cre;TG/wt^ animals showed an impaired PNN structure, with PNNs appearing less dense and almost ruptured (Figure 6).

Additionally, synapse density was analyzed in electron microscopic sections of the CA1 region. Quantification revealed a trend of fewer synapses in mepβ^Cre;TG/wt^ animals compared to their wildtype counterparts. Due to a lack of replicates, no statistical analysis was performed on these sections.

This suggests that cleavage of brevican and RPTPζ in mepβ^Cre;TG/wt^ mice could result in structurally impaired PNNs and reduced synaptic contacts.

## 4 Discussion

Brevican was identified as a proteolytic substrate of meprin β in recombinant soluble and membrane‐associated meprin β homodimers, while meprin α co‐expression possibly enhanced meprin β's catalytic activity (Figures 1, 2, 3). By performing a N‐terminomics analysis comparing mepβ^Cre;TG/wt^ and mepβ^Cre;wt/wt^ mice, the corresponding cleavage sites were mapped at amino acid positions E461_↓_E462, K464_↓_E465, and E584_↓_T585. Additionally, C‐terminal brevican fragments were detected in co‐transfections of HEK293T cells with human brevican and human meprin β (Figure 1A). These fragments had molecular weights of approximately 40 and 60 kDa, respectively. The newly discovered cleavage sites in the murine brevican protein sequence were aligned with the human brevican protein sequence using the NCBI protein BLAST tool [67, 68]. This analysis suggested the presence of correlating cleavage sites in human brevican at E466_↓_E467, E469_↓_E470, and E608_↓_A609. The molecular weights of the resulting unglycosylated C‐terminal fragments were calculated using the ExPASy pI/Mw tool [69] to be 52.1 kDa, 51.74 kDa, and 37 kDa, assuming a full‐length unglycosylated brevican protein weight of 99.12 kDa (Uniprot Database Q96GW7 [54]). Although the amino acid sequences are not totally conserved between human and murine brevican, both fit meprin β's cleavage specificity and fit the size of the C‐terminal fragments observed in the western blots of co‐transfections (Figure 1C). It is worth noting that the anti‐myc tag antibody only detected two fragments in co‐transfections with meprin β. This might indicate a subsequent cleavage at E469_↓_E470 (Figure 1C). Moreover, the unglycosylated molecular weight of the resulting N‐terminal fragments was calculated to appear at 48.25/48.64 and 63.05 kDa using the ExPASy Compute pI/Mw tool [69]. Nevertheless, only fragments of approximately 50 kDa were observed in sMeprin β‐digested cell lysates (Figure 2A). Interestingly, although we observed no statistically significant difference in full‐length brevican levels between cell lysates incubated with sMeprin β and fetuin‐B and those incubated without proteases, N‐terminal fragments were also detected in the cell lysates incubated with sMeprin β and fetuin‐B (Figure 2B). Additionally, there was a non‐significant tendency toward lower brevican protein levels in lysates incubated with sMeprin β and fetuin‐B compared with lysates incubated with mock‐solution (Figure 2C). Taken together, this indicates that fetuin‐B partially inhibits sMeprin β at a molar ratio of 4:1, resulting in an additional approximately 70 kDa fragment (Figure 2B). Thus, these findings suggest that sMeprin β firstly cleaves human brevican at E608_↓_A609, leaving a 70 kDa N‐terminal fragment, and then sequentially cleaves the N‐terminal fragments again at E466_↓_E467 and E469_↓_E470, leaving a 47 kDa fragment. This cleavage would result in additional 23 kDa fragments, which are part of the CS region of the protein. Since the N‐terminal antibody does not detect this region, we were unable to observe these 23 kDa fragments in our western blot analysis (Figure 2A). In summary, our data suggest that human and murine brevican were cleaved by human and murine meprin β, respectively, at conserved cleavage sites.

Neurocan, another component of the ECM and PNNs [9], was identified by the N‐terminomics as an additional probable meprin β substrate. Therefore, we investigated neurocan in co‐transfections with meprin β (Figure 4B). However, we were unable to confirm neurocan cleavage in vitro (Figure 4C), which might be explained by structural differences in the ECM of these cells and the accessibility of the cleavage site. Additionally, neurocan is processed by the signal peptidase in the endoplasmic reticulum at amino acid position G22_↓_D23 [54]. According to the N‐terminomics analysis, the predicted meprin β cleavage sites are located in close proximity to the signal peptidase cleavage site at D26_↓_T27, T27_↓_Q28, and Q28_↓_T29. As western blotting cannot resolve the small difference of six amino acids in full‐length neurocan, which has an approximate unglycosylated molecular weight of 140 kDa [54], significant changes in neurocan protein levels were not expected in this analysis. Due to these methodological limitations, future studies should investigate meprin‐mediated neurocan cleavage using N‐terminal epitope tagging or protein sequencing of cleavage products. Nevertheless, since these cleavage sites were located in the immunoglobulin‐like domain, we propose that essential protein functions, such as binding to hyaluronic acid via its HA link domains, would likely remain intact. Thus, our data suggest that meprin β‐dependent neurocan cleavage may have only minimal effects on ECM structure (Figure 4). It should be noted that neurocan cleavage between T26_↓_Q27, which was attributed to one of the overrepresented peptides, does not match with meprin β's or meprin α's preference for acidic amino acids [44]. Therefore, we hypothesize that an additional protease, likely activated by meprin β, may mediate this cleavage. This would further explain the emergence of neurocan fragments in the HUNTER N‐terminomics analysis.

Together with brevican and neurocan, RPTPζ was also identified as a novel potential proteolytic substrate of meprin β in our HUNTER N‐terminomics analysis. The overrepresented RPTPζ fragments in mepβ^Cre;TG/wt^ animals were generated by cleavage at amino acids E430_↓_E431, which fits both meprin β's and meprin α's cleavage specificity. Concomitantly, in co‐transfections of RPTPζ with meprin β or meprin α, both proteases were shown to cleave RPTPζ (Figure 5B). However, it remains unclear which meprin variant is responsible for the physiological cleavage between amino acids E430_↓_E431. HEK 293 T cells express both meprin α and meprin β endogenously. Endogenous meprin α likely plays a vital role in the cleavage of RPTPζ, as RPTPζ was cleaved when co‐transfected with catalytically inactive meprin β (Figure 5B).

Brevican, neurocan, and RPTPζ, in conjunction with linking proteins like tenascins and HAPLN1, and the glycosaminoglycan hyaluronan, are major structural components of the brain ECM and primarily condense into PNNs around PV+ interneurons [9]. Mepβ^Cre;TG/wt^ mice have previously been observed to have impaired spatial memory and impaired long‐term potentiation [50]. Interestingly, RPTPζ‐knockout mice also exhibited impaired spatial memory in Morris water maze experiments and displayed less dense, attenuated PNNs [65]. RPTPζ is known for its key role in PNN formation, as it tethers PNNs to the cells together with tenascin‐R. This process was reported to enable aggrecan binding, thereby promoting PNN formation [16]. Intriguingly, brevican also plays an essential role in the structural integrity of the brain ECM, learning, and memory formation, as brevican‐knockout mice exhibited impaired CA1 LTP and impaired PNN structure [14]. Furthermore, another known meprin β substrate, tenascin‐C, was recently identified in the intestine [70]. Although tenascin‐C was not analyzed in our HUNTER N‐terminomics experiment, it is also crucial for PNN integrity, as tenascin‐C‐knockout mice again exhibited fewer and structurally impaired PNNs as well [71]. Therefore, PNNs were stained in the hippocampal CA1 region of mepβ^Cre;TG/wt^ and mepβ^Cre;wt/wt^ mice to investigate potential structural alterations by the proteolytic effect of meprins. PNNs appeared less dense in this region (Figure 6). We hypothesize that cleavage of these PNN components and disruption of the PNN structure by meprins in the hippocampal CA1 region contribute to reduced spatial learning in the Morris water maze experiments and impaired CA1 LTP previously shown in mepβ^Cre;TG/wt^ mice [50]. Furthermore, these results suggest that the hippocampus is the central brain region affected, as it is also known to be an essential part in the acquisition of spatial memories [2]. This is supported by the observation of high meprin β activity in the hippocampus of mepβ^Cre;TG/wt^ mice [50]. Thus, our findings suggest that meprin‐mediated ECM remodeling represents a novel mechanism in the pathophysiology of synaptic plasticity and learning.

Synaptic plasticity might indeed be further influenced by meprin‐dependent cleavage of PNN components, as mepβ^Cre;TG/wt^ mice displayed a trend of fewer synapses in the hippocampal CA1 region (Figure 6B). These results are consistent with the finding of diminished inhibitory synapses in combined tenascin C‐, tenascin R‐, brevican‐, and neurocan‐knockout mice [72, 73]. Additionally, excitatory synapse density was diminished in astrocyte‐derived RPTPζ‐knockout mice [64]. We propose that meprin‐mediated digestion of PNN components likely destabilizes synaptic contacts by weakening their structural support in mepβ^Cre;TG/wt^, resulting in a tendency toward lower synapse density. A similar mechanism was hypothesized in mice with kainate‐induced lesions, where proteolytic processing of brevican correlated with synaptic loss [74].

Previous research mainly focused on PNN digestion with ChABC [75]. ChABC enzymatically removes the chondroitin sulfate side chains from PNN proteoglycans, resulting in undetectable PNNs in WFA staining [76]. This digestion of PNNs correlated with impaired recall of remote visually‐induced fear memories [13], impaired auditory fear learning and consolidation [11], enhanced erasure of fear memories [77], and impaired cocaine‐induced place preference memory [12]. Although these studies helped to examine the broader role of PNNs in the brain, PNN digestion with ChABC resembles an unphysiological remodeling of the ECM, as ChABC is not endogenously expressed in mammals. To our knowledge, the proteolytic processing of PNN components, including brevican, neurocan, and RPTPζ, has not been studied in the context of learning and memory. Therefore, we introduce meprin β‐dependent remodeling of the ECM and PNNs as a physiologically relevant and promising target for further investigation into the role of PNNs in neuronal circuit function and pathology.

Beyond ECM proteolysis, meprin β has been shown to cleave APP, forming toxic Aβ species that aggregate into pathologic Aβ plaques, a major hallmark in AD [31, 33]. Interestingly, elevated meprin β mRNA was also found in AD brains [40, 41]. We hypothesize that meprin‐dependent ECM proteolysis may further influence AD pathology, as intact PNNs have been shown to protect against toxic Aβ peptides in vitro [78] and limit the internalization of hyperphosphorylated TAU protein in AD mouse models [79] and human brains [80]. Nevertheless, digestion of PNNs by CHABC was also reported to enhance synaptic plasticity and partially restore object recognition in AD mouse models [75]. To further investigate the physiological relevance of ECM and PNN remodeling in AD, meprin β‐ and APP‐overexpressing mouse models should be studied with a focus on PNN integrity and the effect of enhanced ECM remodeling on synaptic plasticity and learning. Meprin β was also reported as a risk factor for non‐dementia‐related severe cognitive impairments [81]. Upregulation of meprin β in mouse brains impaired spatial memory in the Morris water maze experiments, a finding that could not be attributed to alternative APP processing [50]. Therefore, meprin β‐mediated proteolytic processing of brevican and RPTPζ may contribute to broader cognitive impairments.

In conclusion, this study identifies a meprin‐mediated proteolytic cleavage of the PNN components brevican and RPTPζ, which might impair PNN structure and synaptic plasticity. Therefore, our results highlight meprins as promising targets for the treatment of AD and other forms of non‐dementia‐related cognitive impairments.

## Author Contributions

Claus U. Pietrzik and Simon Kreiselmaier designed the study. Maximilian Keller generated the mouse line. Kira Bickenbach generated the meprin‐knockout cell lines. Constantin Mueller and Simon Kreiselmaier conducted the co‐transfection and western blotting experiments. Nele von Wiegen and Simon Kreiselmaier performed the digestion of brevican with sMeprin β. Mohammad Abukhalaf and Andreas Tholey performed the N‐terminomics experiments and, together with Maximilian Keller and Simon Kreiselmaier, analyzed the data. Simon Kreiselmaier conducted the immunofluorescence stainings. Leonardo Nardi, Maximilian Keller, and Simon Kreiselmaier performed the electron microscopy. Nele von Wiegen, Kira Bickenbach, Leonardo Nardi, and Simon Kreiselmaier wrote the manuscript, which was supervised by Lea Gröbner, Christoph Becker‐Pauly, Kira Bickenbach, Leonardo Nardi, Michael J. Schmeisser, Margareta M. Mueller, and Claus U. Pietrzik.

## Funding

This work was supported by the Deutsche Forschungsgemeinschaft (DFG) CRC 1080 projects A15 (to Claus U. Pietrzik, Christoph Becker‐Pauly) and Z2 (to Andreas Tholey) and Alzheimer Forschungs Initiative Grant 25043R to Claus U. Pietrzik. Project DEAL enabled and organized Open Access funding.

## Ethics Statement

All animal‐involving procedures were carried out in accordance with European and German guidelines for the care and use of laboratory animals. All animal‐involving procedures were approved by the Central Animal Facility of the University of Mainz and the ethical committee on animal care and use of Rhineland–Palatinate, Germany (G20‐1‐026).

## Consent

The final manuscript was read and approved by all authors.

## Conflicts of Interest

The authors declare no conflicts of interest.

## Supporting information


**Data S1:** fsb272097‐sup‐0001‐FiguresS1‐S5.zip.

## Data Availability

All primary data and materials used in this manuscript are available upon reasonable request.
