# Supplementary material for: The Role of Meprins on the Brain Extracellular Matrix and Perineuronal Nets
Source: FASEB J. 2026 Jul 2;40(13):e72097. doi: 10.1096/fj.202601333R (PMC13329426; doi:10.1096/fj.202601333R)
Supplement: Supplementary file 1 — Data S1: fsb272097‐sup‐0001‐FiguresS1‐S5.zip. [file FSB2-40-e72097-s001.zip › 202601333R-sup-0005-SI_Figure-S02.pdf]

# Suppl. Fig. 2

| brevican | | | | |
|---|---|---|---|---|
| log2 fc | master protein accession | master protein description | sequence | positions in master protein |
| -0,556450844 | Q61361 | Brevican core protein OS=Mus musculus GN=Bcan | DDLKEDSSEDR | Q61361 [23-33] |
| -1,171629588 | Q61361 | Brevican core protein OS=Mus musculus GN=Bcan | EDGGGGSSTPEDPAEAPR | Q61361 [407-424] |
| -1,344104131 | Q61361 | Brevican core protein OS=Mus musculus GN=Bcan | GGGGSSTPEDPAEAPR | Q61361 [409-424] |
| -1,102880478 | Q61361 | Brevican core protein OS=Mus musculus GN=Bcan | SSTPEDPAEAPR | Q61361 [413-424] |
| 2,06881396 | Q61361 | Brevican core protein OS=Mus musculus GN=Bcan | EEKEQEDLWVWPR | Q61361 [462-474] |
| 1,133847555 | Q61361 | Brevican core protein OS=Mus musculus GN=Bcan | EQEDLWVWPR | Q61361 [465-474] |
| 2,515839736 | Q61361 | Brevican core protein OS=Mus musculus GN=Bcan | TPSEEKSGR | Q61361 [585-593] |
| -0,206044833 | Q61361 | Brevican core protein OS=Mus musculus GN=Bcan | IYSIPISEDGGGGSSTPEDPAEAPR | Q61361 [400-424] |
| -0,289872487 | Q61361 | Brevican core protein OS=Mus musculus GN=Bcan | YSIPISEDGGGGSSTPEDPAEAPR | Q61361 [401-424] |
| -0,213715235 | Q61361 | Brevican core protein OS=Mus musculus GN=Bcan | SIPISEDGGGGSSTPEDPAEAPR | Q61361 [402-424] |
| 0,224528472 | Q61361 | Brevican core protein OS=Mus musculus GN=Bcan | DGGGGSSTPEDPAEAPR | Q61361 [408-424] |
| -0,476408958 | Q61361 | Brevican core protein OS=Mus musculus GN=Bcan | GSSTPEDPAEAPR | Q61361 [412-424] |
| **neurocan** | | | | |
| 0,863620758 | P55066 | Neurocan core protein OS=Mus musculus GN=Ncan | DTQDTTATEKGLR | P55066 [25-37] |
| 1,27915891 | P55066 | Neurocan core protein OS=Mus musculus GN=Ncan | DTTATEKGLR | P55066 [28-37] |
| 1,16282177 | P55066 | Neurocan core protein OS=Mus musculus GN=Ncan | TTATEKGLR | P55066 [29-37] |
| -0,861163616 | P55066 | Neurocan core protein OS=Mus musculus GN=Ncan | SPDADSIEIEGTSSMR | P55066 [710-725] |
| 0,191365878 | P55066 | Neurocan core protein OS=Mus musculus GN=Ncan | DQDTQDTTATEKGLR | P55066 [23-37] |
| -0,194874128 | P55066 | Neurocan core protein OS=Mus musculus GN=Ncan | EVDEPGAGSLGSR | P55066 [549-561] |
| -0,142034849 | P55066 | Neurocan core protein OS=Mus musculus GN=Ncan | EPGAGSLGSR | P55066 [552-561] |
| **RPTPζ** | | | | |
| -1,199010213 | B9EKR1 | Receptor-type tyrosine-protein phosphatase zeta OS=Mus musculus GN=Ptprz1 | TEEIIKEEEYGKDNEEDTGLNPGR | B9EKR1 [424-447] |
| -0,785587947 | B9EKR1 | Receptor-type tyrosine-protein phosphatase zeta OS=Mus musculus GN=Ptprz1 | IIKEEEYGKDNEEDTGLNPGR | B9EKR1 [427-447] |
| 0,977358659 | B9EKR1 | Receptor-type tyrosine-protein phosphatase zeta OS=Mus musculus GN=Ptprz1 | EEYGKDNEEDTGLNPGR | B9EKR1 [431-447] |
| -0,501127243 | B9EKR1 | Receptor-type tyrosine-protein phosphatase zeta OS=Mus musculus GN=Ptprz1 | DVLKPGSTR | B9EKR1 [628-636] |
| -0,0798165 | B9EKR1 | Receptor-type tyrosine-protein phosphatase zeta OS=Mus musculus GN=Ptprz1 | NEEDTGLNPGR | B9EKR1 [437-447] |
| -0,123798052 | B9EKR1 | Receptor-type tyrosine-protein phosphatase zeta OS=Mus musculus GN=Ptprz1 | EEDTGLNPGR | B9EKR1 [438-447] |
| -0,440167745 | B9EKR1 | Receptor-type tyrosine-protein phosphatase zeta OS=Mus musculus GN=Ptprz1 | TTTHYNHMGTKYNEAKTNR | B9EKR1 [463-481] |
| -0,406127612 | B9EKR1 | Receptor-type tyrosine-protein phosphatase zeta OS=Mus musculus GN=Ptprz1 | STSQHVAEFETER | B9EKR1 [502-514] |
| -0,279747486 | B9EKR1 | Receptor-type tyrosine-protein phosphatase zeta OS=Mus musculus GN=Ptprz1 | ITSSDMPEAITYDVLKPGSTR | B9EKR1 [616-636] |
| 0,106350899 | B9EKR1 | Receptor-type tyrosine-protein phosphatase zeta OS=Mus musculus GN=Ptprz1 | DMPEAITYDVLKPGSTR | B9EKR1 [620-636] |
| -0,460927327 | B9EKR1 | Receptor-type tyrosine-protein phosphatase zeta OS=Mus musculus GN=Ptprz1 | SEAAFSDTASR | B9EKR1 [1156-1166] |
| -0,148319721 | B9EKR1 | Receptor-type tyrosine-protein phosphatase zeta OS=Mus musculus GN=Ptprz1 | ENTEEENGGTGVTR | B9EKR1 [1485-1498] |
| -0,361094475 | B9EKR1 | Receptor-type tyrosine-protein phosphatase zeta OS=Mus musculus GN=Ptprz1 | EKDTDGVLETDDTGIAPGSPR | B9EKR1 [1572-1592] |
| -0,193955421 | B9EKR1 | Receptor-type tyrosine-protein phosphatase zeta OS=Mus musculus GN=Ptprz1 | DTGIAPGSPR | B9EKR1 [1583-1592] |
| 0,053310712 | B9EKR1 | Receptor-type tyrosine-protein phosphatase zeta OS=Mus musculus GN=Ptprz1 | FYLEDNTSPR | B9EKR1 [1668-1677] |
