# Supplementary material for: The Role of Meprins on the Brain Extracellular Matrix and Perineuronal Nets
Source: FASEB J. 2026 Jul 2;40(13):e72097. doi: 10.1096/fj.202601333R (PMC13329426; doi:10.1096/fj.202601333R)
Supplement: Supplementary file 1 — Data S1: fsb272097‐sup‐0001‐FiguresS1‐S5.zip. [file FSB2-40-e72097-s001.zip › Supporting information.docx]

**Supplementary information**

The effect of meprins on the brain extracellular matrix and perineuronal nets

**Supplemental Information**

**Suppl. 1: Genotyping of CRISPR/Cas9 generated HEK293T MEP1A-knockout cells**


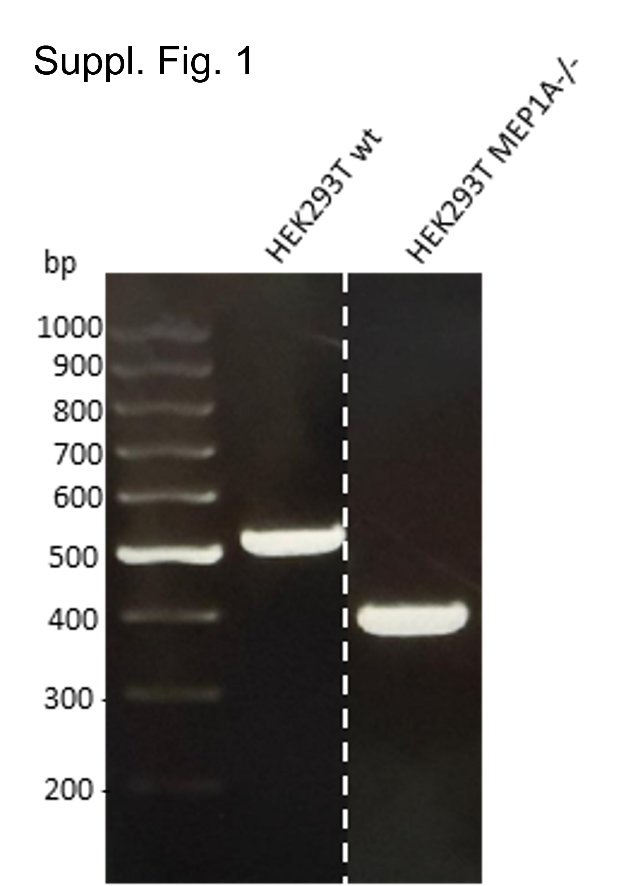


**Suppl. Figure 1.** **Genotype PCRs of HEK293T and CRISPR/Cas9 generated HEK293T MEP1A-knockout cells**. MEP1A-speciﬁc signals were visualized using an agarose gel. A deletion in MEP1A in the HEK293T MEP1A-knockout (HEK293 MEP1A -/-) cells was validated by the size shift of the PCR product.

**Suppl. 2: N-terminomics overrepresented peptides for brevican, neurocan, and RPTPζ**


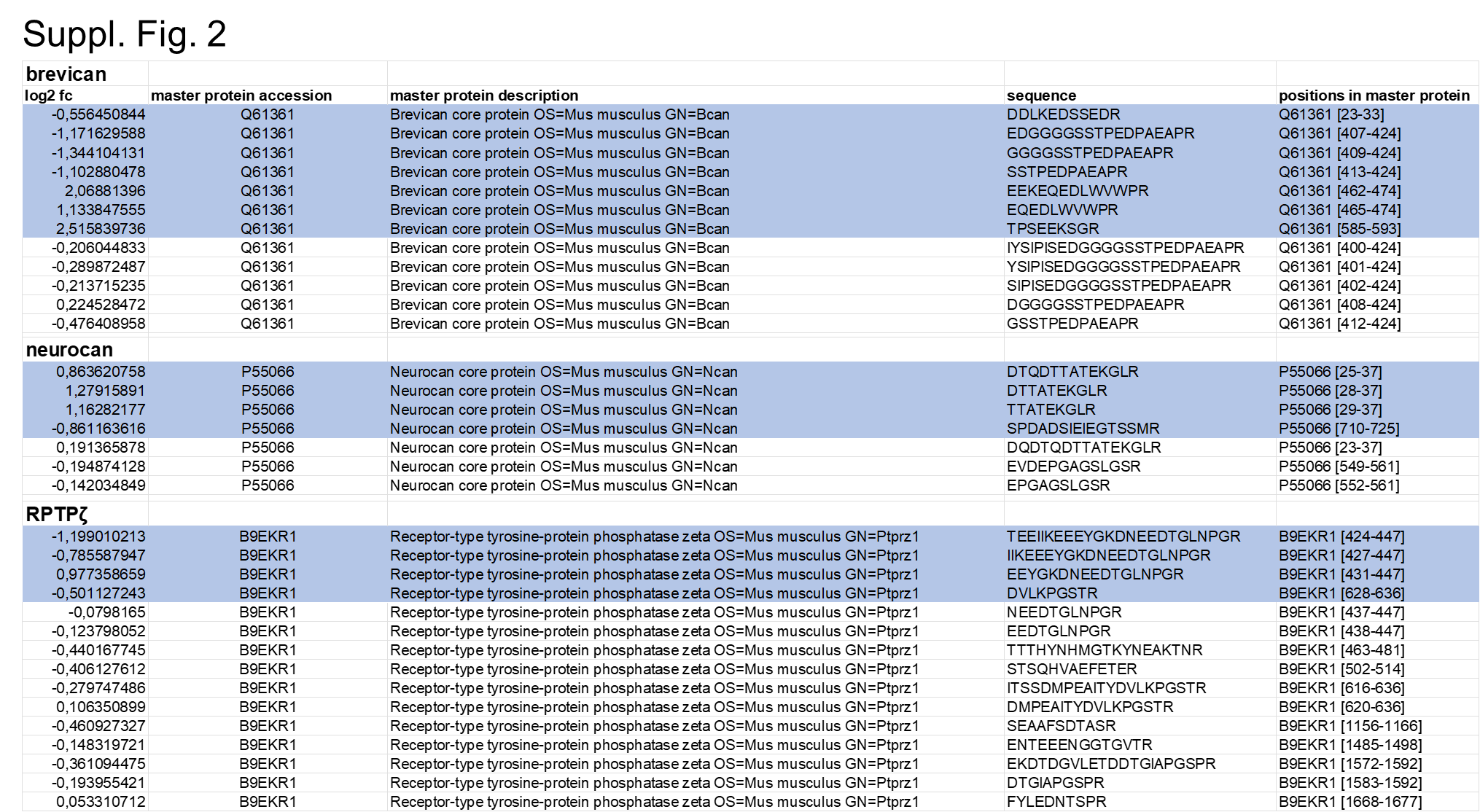


**Suppl. Figure 2. N-terminomics overrepresented peptides for brevican, neurocan, and RPTPζ**. Brevican, neurocan, and RPTPζ N-terminally enriched fragments in mep^Cre;TG/wt^ vs. mep^Cre;wt/wt^ mice. Data is portrayed as a table with columns: log2 fold change, master protein accession, master protein description (name, origin species (OS), gene name (GN)), peptide sequence, and peptide position in the master protein. Peptides are ordered for the master protein, the position in the master protein, and statistical significance. Peptides with a blue background are significantly under- or overexpressed. For peptides with a white background, no statistical significance was detected. Statistical analysis was performed using the permutation-based FDR Student's t-test.

**Suppl. 3: Indirect immunofluorescence of perineuronal nets**


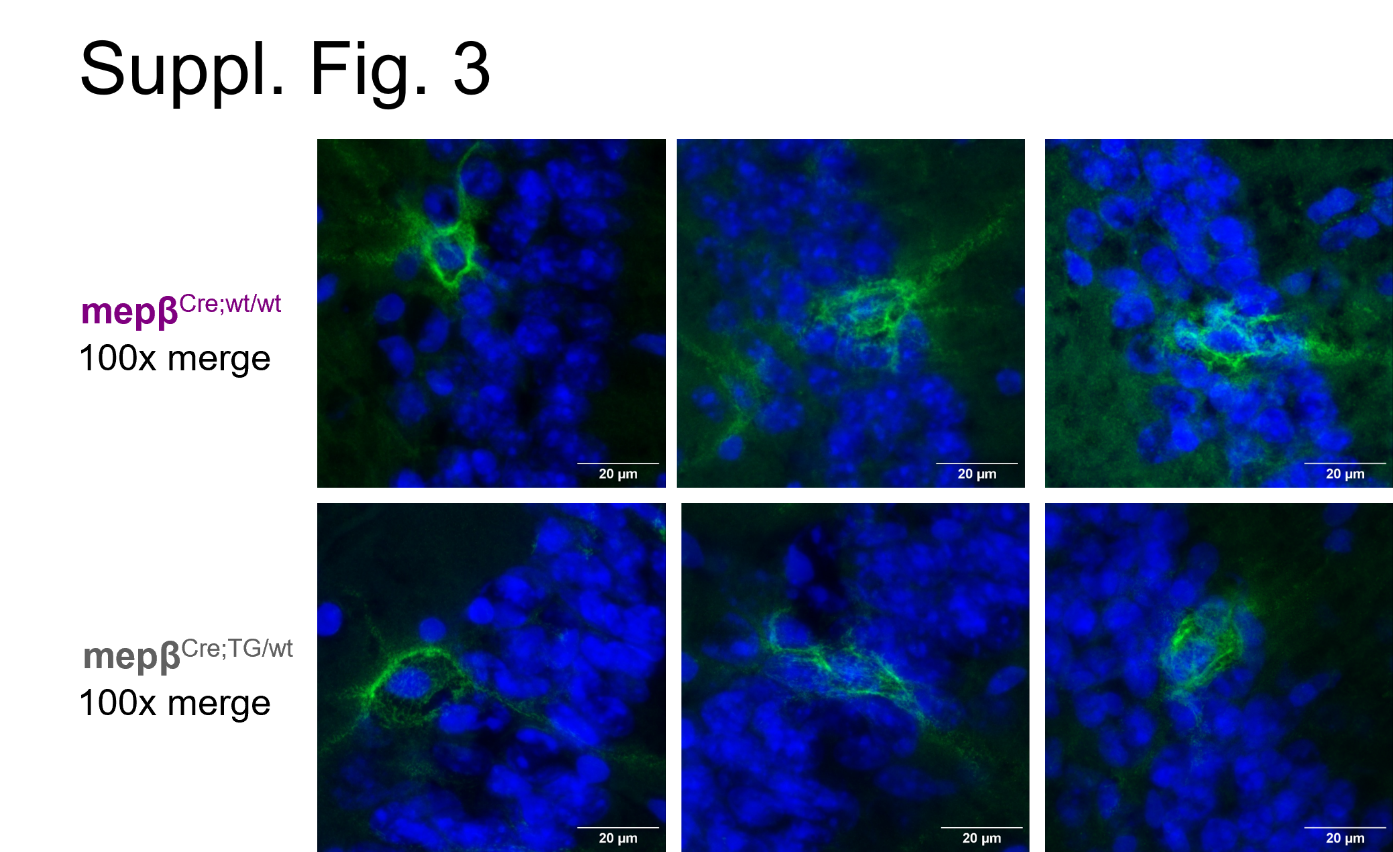


**Suppl. Figure 3. Indirect immunofluorescence of PNNs.** Indirect immunofluorescence images taken o of WFA stained PNNs in the hippocampal stratum pyramidale of the CA1 region. Brain slices were stained with biotinylated WFA with Alexa Fluor 488-coupled streptavidin and DAPI. Slices from 10 month-old mepβ^Cre;wt/wt^ and mepβ^Cre;TG/wt^ mice were compared. PNNs appeared structurally impaired in mepβ^Cre;TG/wt^ mice.

**Suppl. 4: Schematic illustration of meprin variants**


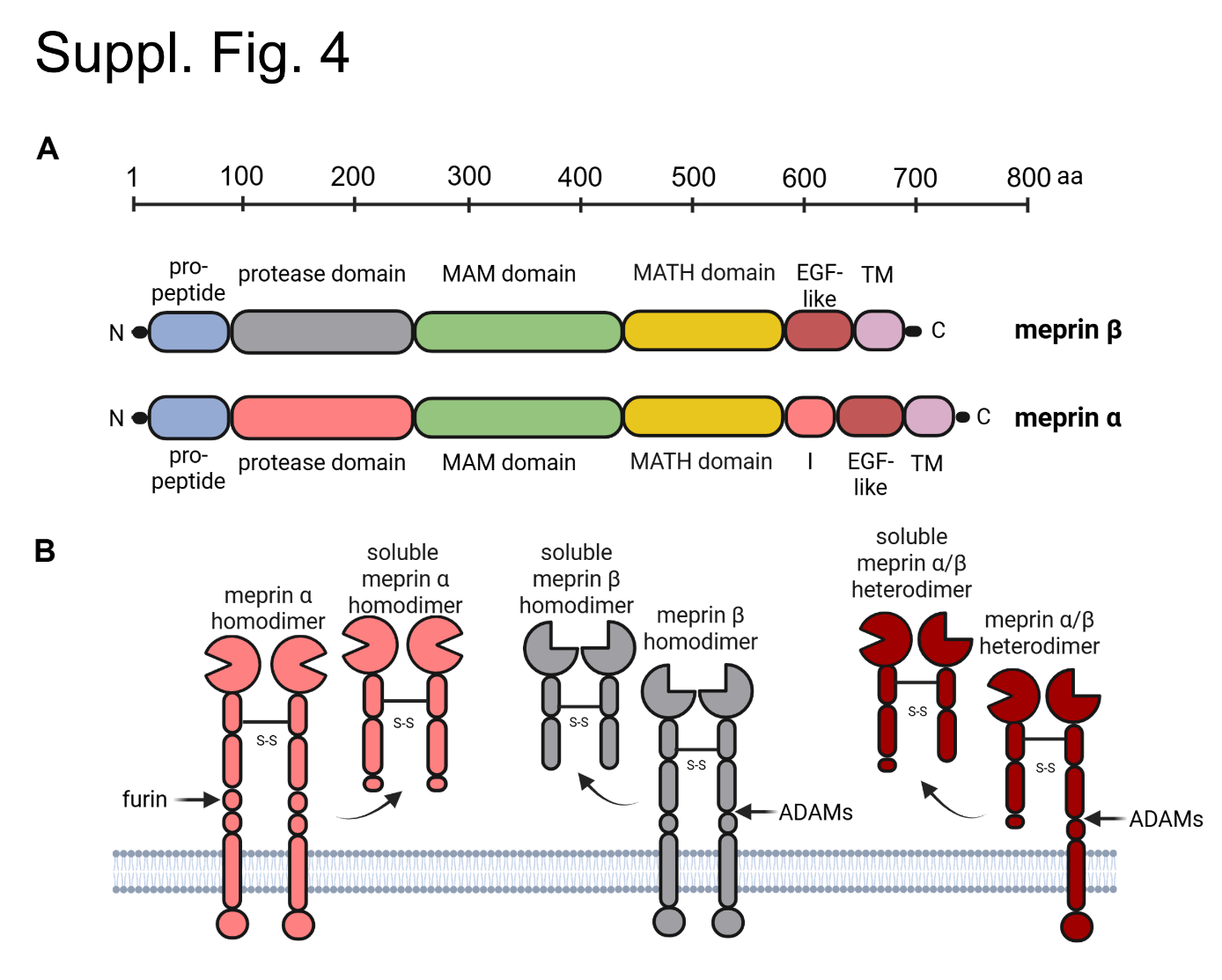


**Suppl. Figure 4. Schematic illustration of meprin variants. (A)** Schematic illustration of the protein structure of meprin β and α. Both meprins consist of a N-terminal pro-peptide, a protease domain, a meprin A5 protein and receptor protein tyrosine phosphatase mu (MAM) domain, a meprin and TRAF homology (MATH) domain, an epidermal growth factor (EGF)-like domain, and a transmembrane (TM) domain. Additionally, meprin α contains an inserted domain (I) that includes a furin cleavage site. **(B)** Schematic illustration of meprins in homodimers and heterodimers. Meprin α is secreted by furin cleavage in the inserted domain. Meprin β is secreted by ADAM 10/17 cleavage N-terminal of its EGF-like domain. Meprin β can only be secreted as a zymogen. Meprin α/β heterodimers can also be shed from the membrane by furin and ADAM 10/17.

**Cell surface biotinylation assay**


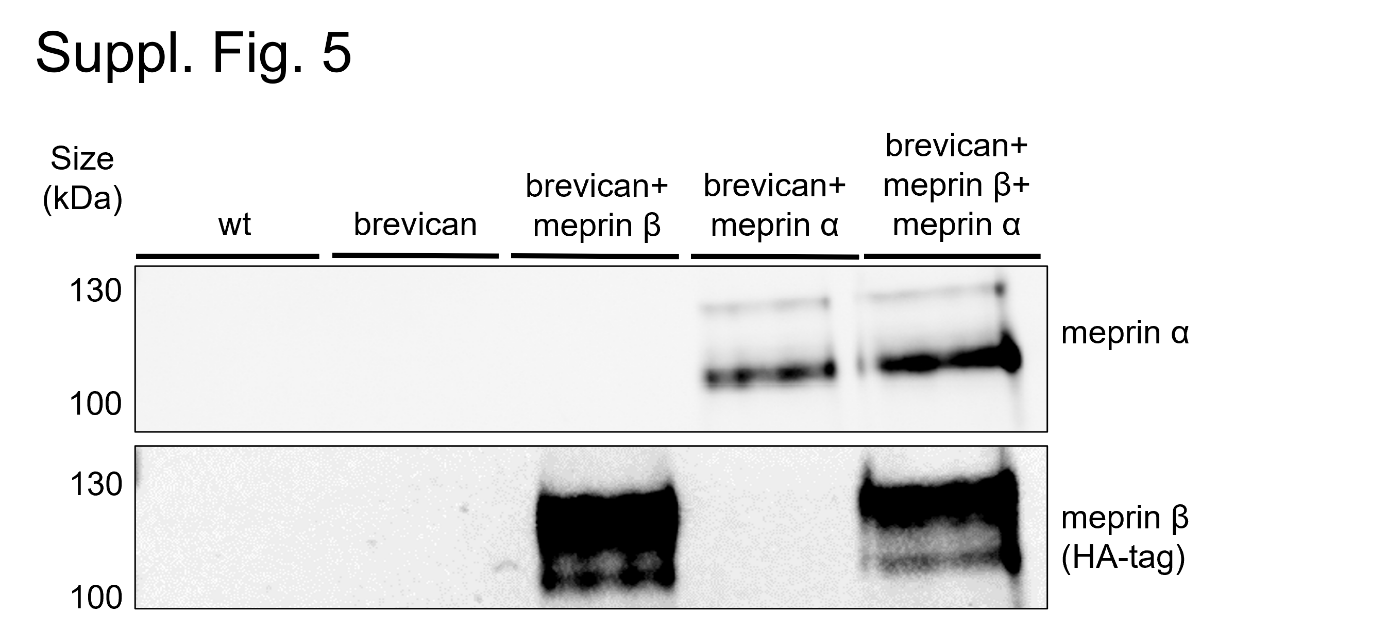


**Suppl. Fig. 5. Cell surface biotinylation assay.** To assess if meprin α can be located at the cell surface in triple transfections, HEK 293T wt cells were triple transfected with brevican, meprin β, and/or meprin α. Then, cell surface proteins were biotinylated and pulled down for western blot analysis. This Fig. shows a representative western blot of meprin α and meprin β at the cell surface. Notably, in co-transfections with meprin β, elevated meprin α levels were detected at the cell surface. This indicates that heterodimerization, as shown by other groups [49], exists in our setup, is enhanced by co-transfection of meprin β and meprin α, and indeed, tethers meprin α in its furin-cleaved form (approximately 100kDa) to the cell surface.
