# Supplementary figures and images for: The Role of Meprins on the Brain Extracellular Matrix and Perineuronal Nets
Source: FASEB J. 2026 Jul 2;40(13):e72097. doi: 10.1096/fj.202601333R (PMC13329426; doi:10.1096/fj.202601333R)

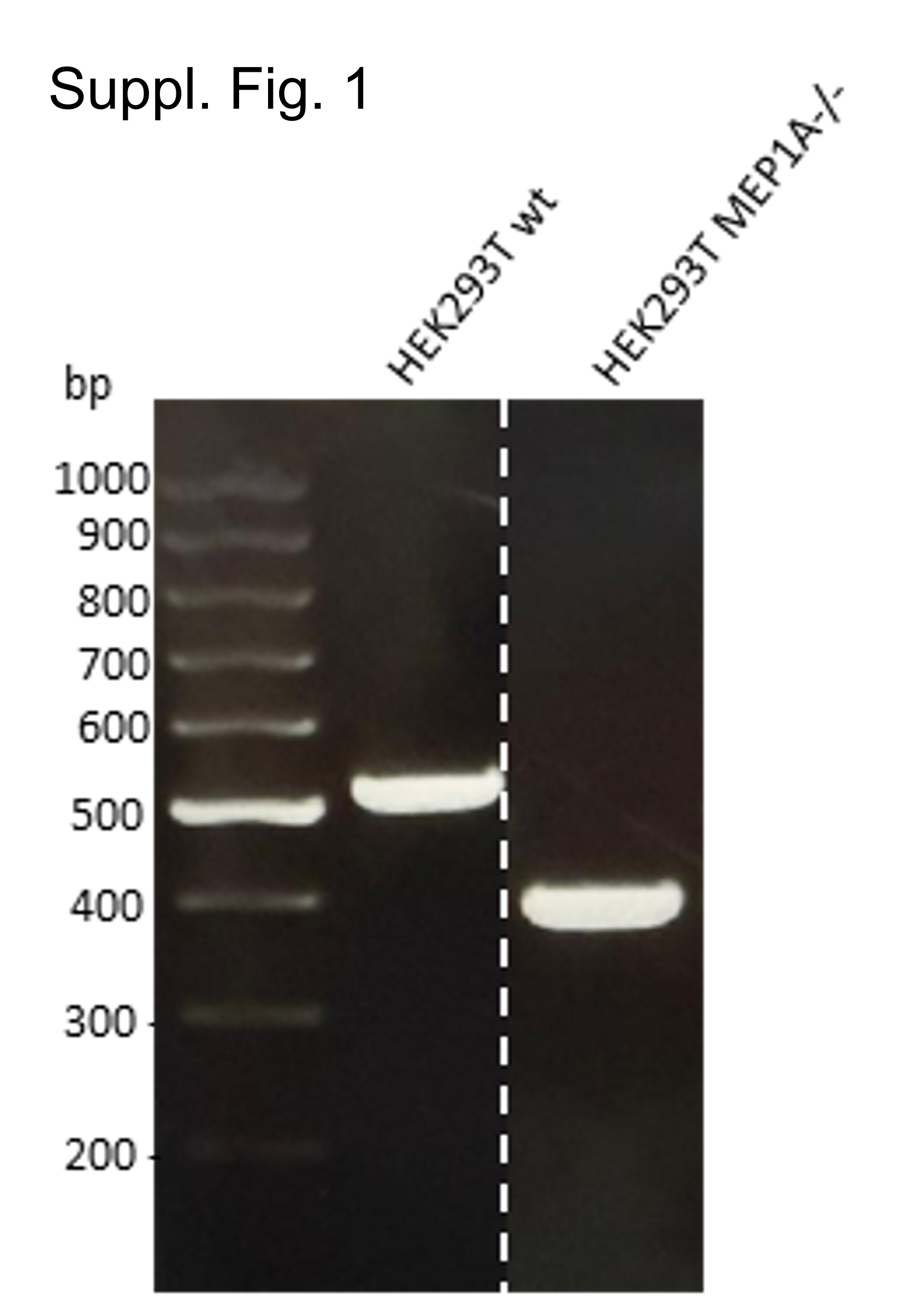

Supplement: Supplementary file 1 — Data S1: fsb272097‐sup‐0001‐FiguresS1‐S5.zip. [file FSB2-40-e72097-s001.zip › 202601333R-sup-0004-SI_Figure-S01.tif]

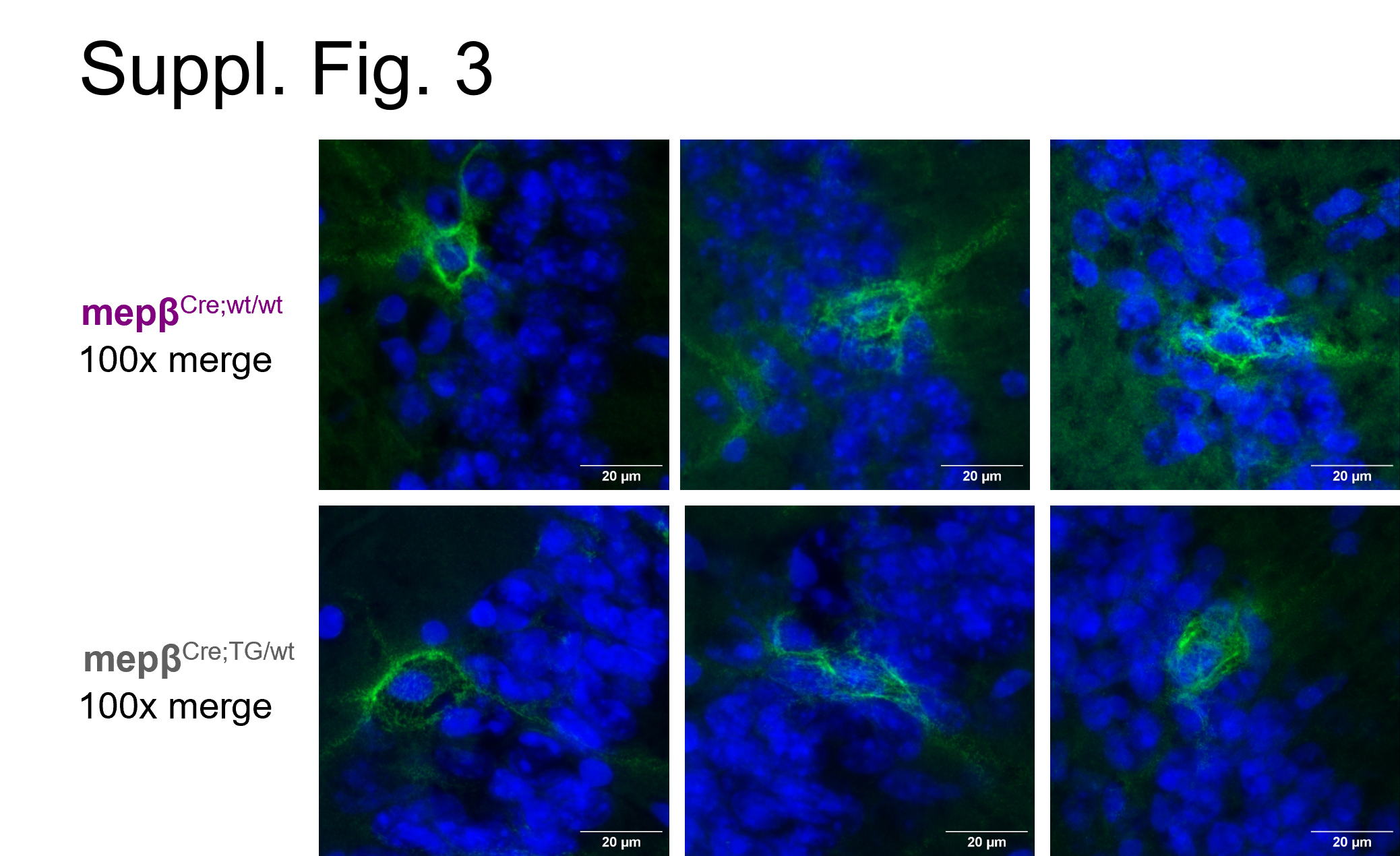

Supplement: Supplementary file 1 — Data S1: fsb272097‐sup‐0001‐FiguresS1‐S5.zip. [file FSB2-40-e72097-s001.zip › 202601333R-sup-0006-SI_Figure-S03.tif]

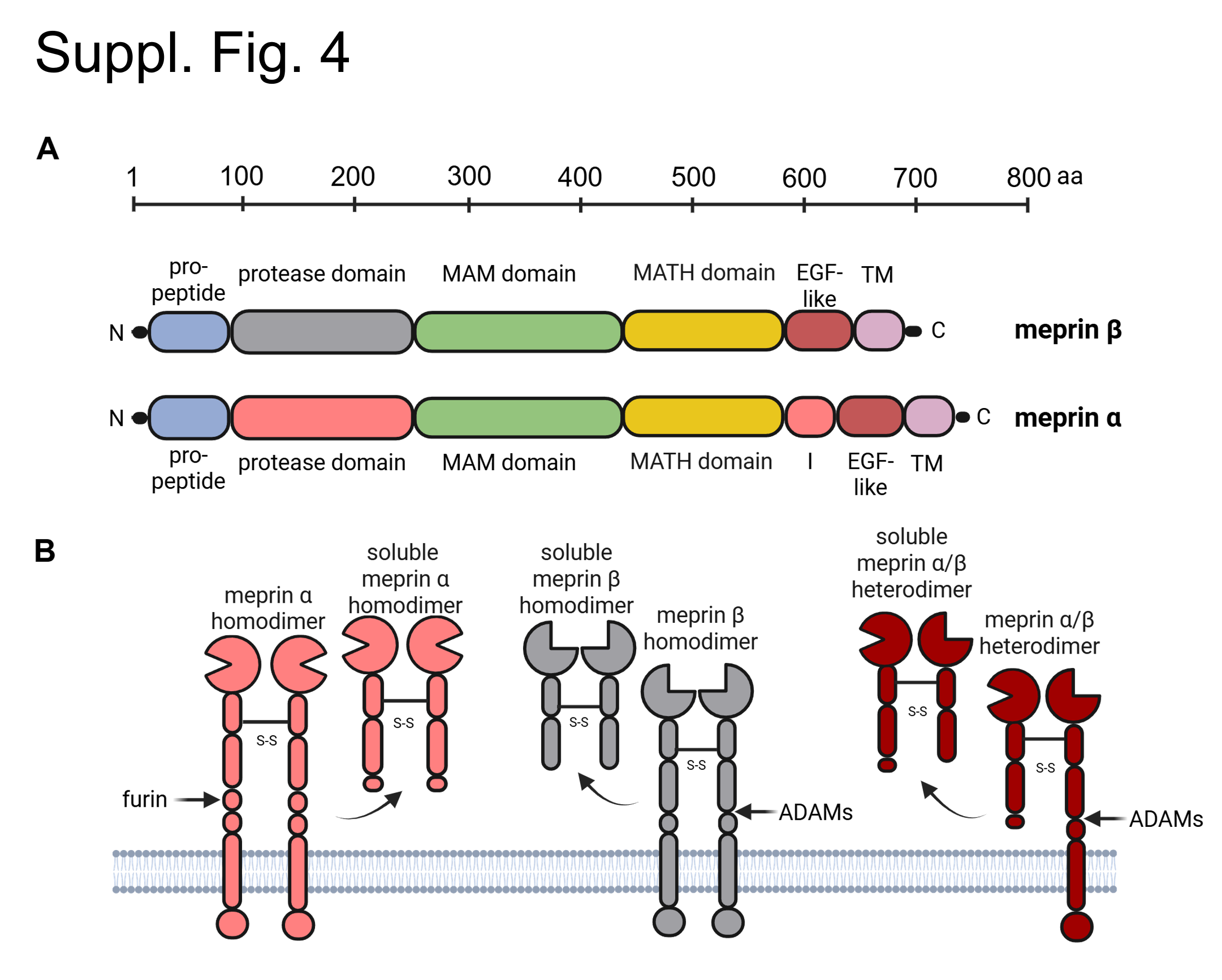

Supplement: Supplementary file 1 — Data S1: fsb272097‐sup‐0001‐FiguresS1‐S5.zip. [file FSB2-40-e72097-s001.zip › 202601333R-sup-0007-SI_Figure-S04.tif]

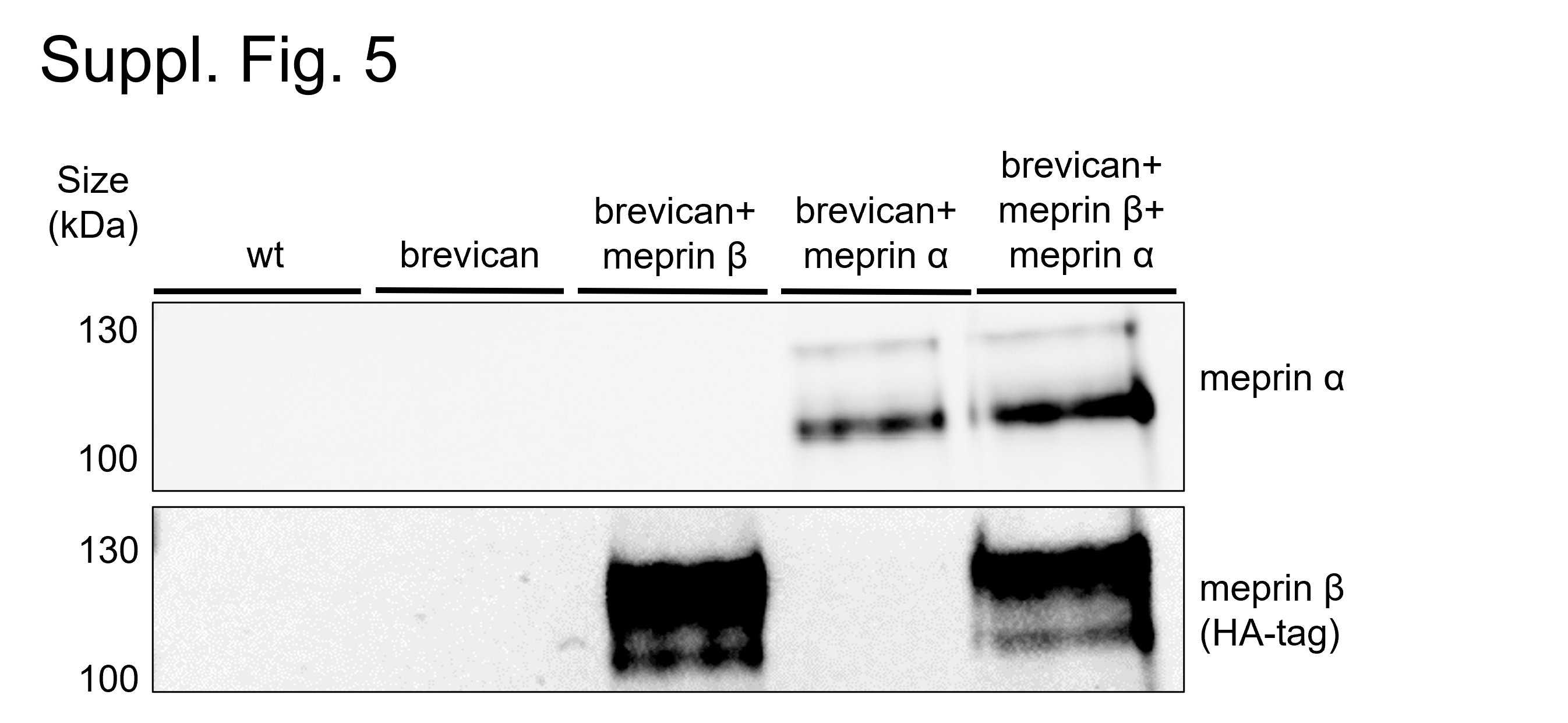

Supplement: Supplementary file 1 — Data S1: fsb272097‐sup‐0001‐FiguresS1‐S5.zip. [file FSB2-40-e72097-s001.zip › 202601333R-sup-0008-SI_Figure-S05.tif]
